# Molecular-Level Release of Coumarin-3-Carboxylic Acid and Warfarin-Derivatives from BSA-Based Hydrogels

**DOI:** 10.3390/pharmaceutics13101661

**Published:** 2021-10-11

**Authors:** Niuosha Sanaeifar, Karsten Mäder, Dariush Hinderberger

**Affiliations:** 1 Institute of Chemistry, Martin Luther University Halle-Wittenberg, Von-Danckelmann-Platz 4, 06120 Halle (Saale), Germany; niuosha.sanaeifar@chemie.uni-halle.de; 2 Institute of Pharmacy, Martin Luther University Halle-Wittenberg, Wolfgang-Langenbeck-Str. 4, 06120 Halle (Saale), Germany; karsten.maeder@pharmazie.uni-halle.de

**Keywords:** albumin, hydrogels, EPR/ESR spectroscopy, release behavior

## Abstract

This investigation aimed at developing BSA hydrogels as a controlled release system to study the release behavior of spin-labeled coumarin-3-carboxylic acid (SL-CCS) and warfarin (SL-WFR). The release profiles of these spin-labeled (SL-) pharmaceuticals from BSA hydrogels prepared with different procedures are compared in detail. The mechanical properties of the gels during formation and release were studied via rheology, while a nanoscopic view on the release behavior was achieved by analyzing SL-drugs–BSA interaction using continuous wave electron paramagnetic resonance (CW EPR) spectroscopy. The influence of type of drug, drug concentration, duration of gel formation, and gelation methods on release behavior were characterized by CW EPR spectroscopy, EPR imaging (EPRI), and dynamic light scattering (DLS), which provide information on the interaction of BSA with SL-drugs, the percentage of drug inside the hydrogel and the nature and size of the released structures, respectively. We found that the release rate of SL-CCS and SL-WFR from BSA hydrogels is tunable through drug ratios, hydrogel incubation time and gelation procedures. All of the results indicate that BSA hydrogels can be potentially exploited in controlled drug delivery applications.

## 1. Introduction

Conventional drug administration leads to an elevated drug concentration in the blood followed by a drop until the next dosage is administered [1]. The administration of a single large dose causes the drug level to rise above the minimum toxic concentration (MTC), leading to harmful side effects, and then rapidly decreases below the minimum effective concentration (MEC). Frequent dosing may maintain the drug level within the therapeutic range, however, it can decrease patient compliance [2]. Controlled drug delivery systems in which the entire therapeutic dose is administered at once can avoid high fluctuations of the drug plasma level, minimize possible side effects, and release the drug in a well-defined behavior over extended periods of time [3]. Various systems have been developed as controlled drug delivery systems such as polylactic acid (PLA) and poly(lactic-*co*-glycolic acid) (PLGA) products [4,5], nanocarriers [6,7], smart polymers [8,9], hydroxyapatite (HA) [10], hydrogels [11,12,13], and others.

Hydrogels are entangled networks made from proteins, hydrophilic polymers, or small molecules with the capability to retain water and biological fluids in large amounts without dissolving, due to the presence of physical and chemical bonds between the polymeric chains [14,15,16]. Their swollen, soft, and rubbery nature resembles living tissues and minimizes negative immune reaction after implantation [14,17]. Since a drug can be incorporated in the water-swollen network and released gradually, hydrogels have emerged as excellent candidates for the controlled release of therapeutics [18].

**Figure 1:**
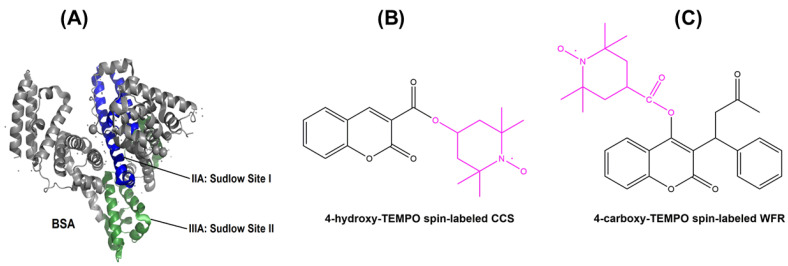
(**A**) Crystal structure of BSA and its drug-binding sites (PDB ID: 4f5s) and molecular structures of (**B**) SL-CCS and (**C**) SL-WFR.

In order to develop a novel drug delivery vehicle, we selected bovine serum albumin (see Figure 1A) as our drug carrier because of its high biocompatibility, availability, stability, and low cost [19,20]. Although albumins do show species-specific differences in solution structure and dynamics [21,22,23], from a general perspective, serum albumin is a heart-shaped molecule in the crystalline state, and the most abundant protein in the circulatory system of vertebrates [24]. In addition to maintaining osmotic pressure and blood pH, it transports a variety of ligands such as un-esterified fatty acids, hormones, proteins, and drug compounds to different tissues through physical or chemical bonding to the binding sites of this protein [17,25,26].

The mechanism of hydrogel formation from albumin by heating and chemical crosslinking has been previously established [27]. Thermally induced gel formation requires conformational changes and unfolding of polypeptide segments induced by heating above denaturation temperature of the albumin, which results in the availability of functional groups present in intramolecular hydrogen bonding for intermolecular interactions, which are essential in the aggregation and build-up of the gel network [28]. Albumin hydrogels can be generated at 37 °C by lowering the pH to 3.5. In these pH-triggered gels, changes in the net charge of the protein from −16 mV at pH 7.4 to +100 mV at pH 3.5 cause the repulsion of protein domains and subsequent partial denaturation [29]. We recently elucidated the ability to form hydrogels from bovine and human serum albumin below their denaturation temperature and wide pH range [19]. Moreover, ethanol induced hydrogels can be formed at 37 °C by mixing different concentrations of BSA solution and chemical denaturant ethanol [27].

Several reports are available on the use of albumin for drug delivery purposes, but they have been largely based on nanocarrier systems. For instance, Pápay et al. studied the pulmonary delivery of apigenin, a natural polyphenol with antioxidant activity from BSA nanoparticles [30]. Gharbavi et al. developed a microemulsion system based on BSA nanoparticles to investigate the release behavior of paclitaxel and folate [31]. In another experiment by de Redín et al., human serum albumin nanoparticles were prepared to explore the ocular delivery of Bevacizumab [32].

Electron paramagnetic resonance (EPR) spectroscopy is a non-destructive and highly specific method used to monitor paramagnetic compounds containing unpaired electrons. The majority of materials, except for paramagnetic transition metal ions that possess intrinsic free radicals, are not detectable by EPR due to the absence of paramagnetic centers. Thus, by spin labeling or spin probing techniques, which are based on the covalent or non-covalent corporation of paramagnetic substances such as organic nitroxide radicals into the drug, the protein, or both, it is possible to study drug–protein interactions in drug delivery systems. Furthermore, through this method, information about the local environment, motional parameters, and ligand binding of the protein can be obtained, which allow for deep insights into the release properties [16,25,27,33]. Spectral information as well as spatial distribution of free radicals can be studied by EPR imaging. Thus, this method has been implemented as a useful technique to probe property changes in different parts of an object that releases drugs [34,35].

Coumarin (CCS) and warfarin (WFR) are widely prescribed as anticoagulant drugs for the treatment of thromboembolic disorders, which are among the most common and often fatal cardiovascular diseases. Moving toward a more controlled delivery of these types of drugs can avoid problems related to their oral administration including variability in dose response due to their narrow therapeutic window or considerable interaction with other medications [36,37,38]. Previously, our group synthesized various spin-labeled pharmaceuticals (SLP) including warfarin and coumarin-3-carboxylic acid (see Figure 1B,C) and analyzed drug binding to human serum albumin in solution with serum-like concentrations via the continuous-wave EPR spectroscopic approach [39]. Furthermore, the release of 16-doxyl stearic acid (16-DSA) as a model drug from BSA hydrogels and the nanoscopic properties of released components has been studied extensively [20]. In the present report, we aimed at developing and comparing controlled delivery systems based on BSA hydrogels and spin-labeled coumarin-3-carboxylic acid (SL-CCS) and warfarin (SL-WFR) as our candidate drugs to investigate the release behavior from the prepared gels. The parameters taken into consideration were the method of gel formation, the drug concentration, and the incubation time, which is the time required for gelation to process at specific temperature or pH. Our investigation is complemented by studying the influence of these parameters on the mechanical properties of the gels using rheology, the interaction and spatial distribution of SL-drugs with BSA gels by means of CW EPR spectroscopy and EPR imaging, and the size and nature of released components by DLS. From the combined data, we finally draw conclusions on the release mechanisms from the gels.

## 2. Experimental Section

### 2.1. Materials

Bovine serum albumin (fatty acid free, lyophilized powder, BSA > 96%) was purchased from Sigma-Aldrich (Taufkirchen, Germany) and used as received without any purification. 4-Hydroxy TEMPO-labeled coumarin-3-carboxylic acid (SL-CCS) and 4-carboxy-TEMPO-labeled warfarin (SL-WFR), synthesized previously [39], were used in this research. In our recent work [20], we introduced a concise notation, BSA_i_(θ, p, t, r), to describe the gelation technique (i), which can be either thermally (T) or pH (P) induced methods, temperature (θ) and pH (p) of hydrogel preparation, incubation time (t, in minutes), and time-release (r, in hours) indicating the release of drug over a specific time, respectively. For instance, BSA_T_ (65, 7.4, 5, 48) refers to the thermally prepared gel at 65 °C for 5 min and pH 7.4, and the release behavior was studied 48 h after gelation.

### 2.2. Methods

The 3 mM precursor solution of BSA was prepared by dissolving BSA powder in deionized water. After 1 h of stirring at 100 rpm at room temperature, the final solution was sterilized by a 0.45 µm filter. SL-CCS and SL-WFR powders were dissolved separately in DMSO to obtain 26 mM stock solutions and added into the BSA precursor solution with three different drug:BSA molar ratios into glass vials. Hydrogels were prepared by adopting the procedures described in our paper [20]. Thermally induced gels were obtained by keeping the vials in a thermomixer at 65 °C and 59 °C, above and below the denaturation temperature of BSA, while some hydrogels were prepared at 37 °C by the addition of 2 M HCl and lowering the pH to 3.5 by the so-called pH induced method.

### 2.3. In Vitro Drug Release

Our release experiment was based on the addition of 1 mL 10× PBS on top of the hydrogels and incubating vials in a thermomixer at 37 °C. In order to maintain sink condition, after removal of 12 µL release medium at various time intervals for deeper analysis, this amount was replaced with fresh PBS. 

### 2.4. Rheological Characterization

The mechanical properties and viscoelastic behavior during the formation of the hydrogels were investigated using rheological characterization. Evaluation of storage and loss moduli (G′ and G″, respectively) provides information on gelation point (i.e., the start-time for deviation of G′ from G″). According to the shape and magnitude of storage and loss moduli, it is possible to obtain an insight into the gel stability and define mechanically robust and weak hydrogels.

Rheological measurements of hydrogels were performed on a Physica MCR 301 rheometer (Anton Paar, Graz, Austria) using a CP50-2/TG plate as a measurement system. After placing the 2 mL of precursor solutions, namely BSA and different ratios of CCS/WFR:BSA, on the surface of the rheometer plate, the measurement gap was covered by silicon oil to avoid water evaporation. Time dependent curves of storage and loss moduli during gel formation were obtained using a 0.5% oscillatory strain and 1 rad s^−1^ frequency.

### 2.5. Continuous Wave Electron Paramagnetic Resonance (CW EPR) Spectroscopy

CW EPR spectroscopy provides information on the release mechanisms by plotting the double integral of the released spin-labeled drugs in the EPR spectra versus release time intervals, which is a measure of the released molecules. Furthermore, a deeper understanding of the drug–protein interaction in the hydrogel and the release medium can be obtained by analyzing the rotational motion of the spin probe. For instance, immobilized radicals show slow rotational dynamics that are coupled to the much slower rotational motion of the protein, while freely tumbling spin probes show no or little attachment to proteins.

The CW EPR measurements were conducted on a Miniscope MS400 (Magnettech, Berlin, Germany) benchtop spectrometer with a microwave frequency of 9.4 GHz recorded with a frequency counter (Racal Dana 2101, Neu-Isenburg, Germany). The temperature of the samples was set to 37 °C using temperature controller H03 (Magnettech). All experiments were performed at a microwave power of 15 mW, a sweep width of 15 mT, and modulation amplitude of 0.2 or 0.03 mT (for analyzing release medium and hydrogels, respectively). Simulations of recorded EPR spectra were evaluated in MATLAB using the EasySpin program package. This program was based on applying the Schneider–Freed approach to solve the Schrödinger equation for slowly rotating nitroxides [40].

Sample preparation was as follows: about 12 µL of desired solution were filled into 50 µL glass capillary (Blaubrand, Wertheim, Germany) and capped with tube sealant (Leica Critoseal, Wetzlar, Germany).

### 2.6. Electron Paramagnetic Resonance Imaging

Spectral-spatial electron paramagnetic resonance imaging (EPRI) is a well-suited technique to reveal a spatial tomogram as well as an EPR spectrum. The former derives information on the macromolecular structure of the sample while the latter indicates the micromolecular structure arises from mobility of the paramagnetic center [41].

In this work, EPRI was used to monitor the release procedure by analyzing hydrogels, while the released substances in the medium were investigated by applying CW EPR.

The EPRI spectra were obtained using an L-band spectrometer (Magnettech, Berlin, Germany) equipped with a re-entrant resonator, operating at a microwave frequency of about 1.1–1.3 GHz.

The measurement parameters used for 2D spatial imaging were set as follows: center field 48.8 mT, scan width 8 mT, scan time 60 s, and modulation amplitude 0.02 mT.

The samples were prepared by placing 600 µL of precursor solution into cylindrical Teflon sample holders (9.3 mm diameters and 8.76 mm height) and keeping them in an oven (UE 400, Memmert, Büchenbach, Germany) with different incubation times. After gel formation, sample holders were placed into 100 mL flasks containing 65 mL 10× PBS and kept in an incubation shaker at 37 °C. For EPRI measurements, at different time intervals, the sample holders were taken out of the buffer, externally dried with a tissue and subsequently transferred into the spectrometer.

### 2.7. Dynamic Light Scattering

Dynamic light scattering (DLS) measures fluctuations of the scattering intensity arising from Brownian motion of particles in solution. By analyzing these fluctuations, information about the diffusion correlation and particle size can be obtained. DLS is one of the most popular techniques used as a non-destructive method to characterize complex liquids, proteins, and polymer structures [20,42,43].

A Litesizer 500 (Anton Paar GmbH, Graz, Austria) equipped with a 40 nm semiconductor laser with a wavelength of 658 nm was used for these experiments. The instrument measures with six runs and the duration of 30 s for each run and the temperature was set to 37 °C (with 4 min of equilibration time).

To obtain the hydrodynamic radius and intensity correlation function, the release medium was analyzed by transferring 100 µL of release sample into a Quartz cuvette (Hellma Analytics) with no further filtration. Thereafter, the samples were measured to gain insights into the nature of the released molecules and gel fragments.

## 3. Results and Discussion

### 3.1. Rheological Measurement

Mechanical and viscoelastic properties of hydrogels can be considered essential design parameters for pharmaceutical and biomedical applications. In the field of drug delivery, the integrity of the hydrogel plays a crucial role in protecting a therapeutic agent in the case of a non-biodegradable system until it is released out of the system, while the flow properties of some gels gain importance since they are used as injectable carriers for drug delivery purposes [44,45].

**Figure 2:**
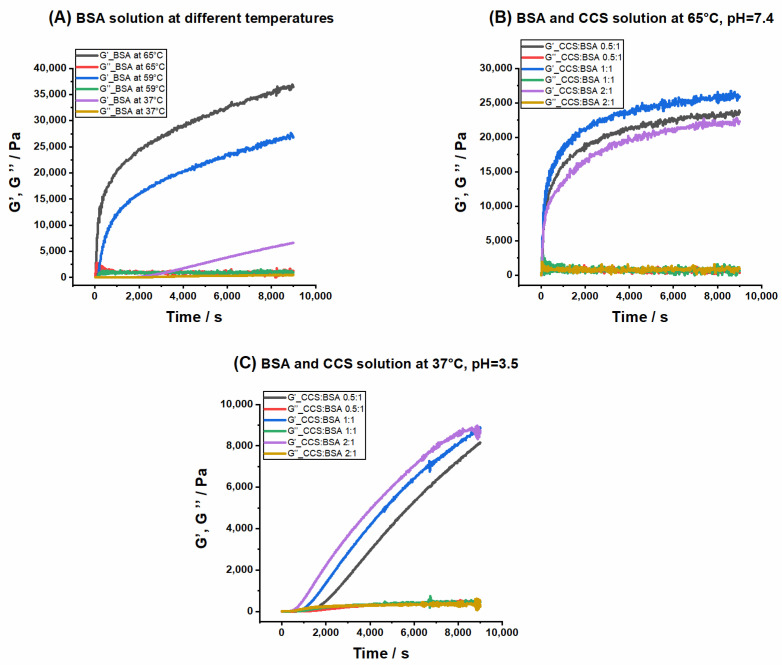
Storage (G′) and loss (G″) moduli as a function of time for (**A**) BSA_T_(65, 7.4), BSA_T_(59, 7.4), and BSA_p_(37, 3.5); (**B**) different ratios of CCS:BSA at 65 °C, pH 7.4; and (**C**) different ratios of CCS:BSA at 37 °C, pH 3.5.

Physical protein gels are formed by non-covalent interchain associations involving hydrophobic, van der Waals, solvation, and electrostatic interactions determining the rheological behavior of proteins. The rigidity of the protein network arises from the strength and number as well as pattern of these interactions [19,46]. Figure 2A shows the time dependent storage (G′) and loss (G″) moduli of 3 mM BSA precursor solution at different temperatures.

For thermally induced hydrogels, the precursor solutions of BSA were heated and kept at 65 °C and 59 °C (slightly above and below the denaturation temperature of BSA, respectively). In both samples, the storage modulus dominated the loss modulus instantly, reflecting the gelation point. We defined a hydrogel to be mechanically tough when the G′ value exceeded 10,000 Pa after 1 h. The value of G′ for BSA_T_(65, 7.4, 60) and BSA_T_(59, 7.4, 60) increased up to 29,000 and 20,000 Pa after 60 min, respectively. Therefore, robust hydrogels can be formed by the heat induced technique even at 59 °C, which is slightly below the denaturation temperature of BSA. However, the G′ value of BSA_T_(65, 7.4) was, as expected, considerably higher than that of the BSA_T_(59, 7.4). In our previous work [20], we extensively studied changes in the secondary structure of BSA during gelation.

In Figure 2A, we also present gel formation (i.e., rheology at 37 °C) through the addition of 2 M HCl to a BSA precursor solution, which reduces the pH to 3.5. Gelation in this hydrogel sets in after 25 min, and is less rigid compared to hydrogels formed by heating. The gelation rate in pH-triggered hydrogels is relatively slow, so that in order to obtain mechanically tough hydrogels, much longer incubation times are required.

To probe the impact of CCS and WFR on albumin gelation kinetics, different ratios of these drugs were added to the precursor solutions of BSA and rheological measurements were repeated at different temperatures (see Figure S1 for CCS:BSA with different molar ratios at 59 °C). Due to the similarity in mechanical properties of WFR with CCS, the whole rheological characterization of WFR is explained in detail in the Supplementary Materials. Human serum albumin (HSA) has two main drug binding sites called Sudlow sites I and II, located in the hydrophobic cavities of subdomains IIA and IIIA, respectively. Sudlow site I can bind bulky heterocyclic compounds, while site II has a high binding affinity to aromatic compounds [47,48]. According to [39], HSA has only one high affinity binding site for SL-WFR, while SL-CCS occupied three binding sites of this protein, but with lower affinity. It is important to note that the binding characteristics of BSA show high resemblance to that of HSA [49].

Time dependence of storage and loss moduli for the system including BSA and CCS with three different molar ratios at 65 °C are depicted in Figure 2B. It seems that the addition of CCS leads to a lower storage modulus (around 10,000 Pa) and weaker mechanical properties compared to the BSA hydrogel formed at the same temperature without CCS. This is due to the presence of CCS in the Sudlow binding sites in albumin (see Figure 1A), which stabilize BSA structure to some extent and thus impede the conformational changes. Similar effects are known for bound fatty acids [22,50]. Apparently, the concentration of CCS cannot deeply affect thermally-induced gelation, since the storage modulus values for three different ratios are similar. However, pH-triggered hydrogels demonstrate the opposite behavior (see Figure 2C). Gelation starts faster and the storage modulus increases slightly when CCS is added to the BSA precursor solution. As explained in the CW EPR section, the percentage of bound SL-CCR to BSA in pH-induced hydrogels is very low. It seems that CCS does not fully bind to Sudlow sites with this preparation method and instead may interact with the surface of the protein, which may facilitate denaturation and may then result in the observed earlier gelation starting point.

We have previously investigated the effect of fatty acids on gel formation by adding different concentrations of stearic acid (SA) to a BSA precursor solution (SA and 16-DSA are substitutable in their binding to BSA). Unlike CCS and WFR, fatty acids delay gelation onset time and considerably affect the mechanical properties. Generally, BSA consists of seven long chain fatty acid binding sites that obstruct protein denaturation and final gelation and increase the thermal stability of protein [20].

### 3.2. Release Studies of SL-CCS and SL-WFR Loaded BSA Hydrogels and Gel Characterization

Drug binding to HSA in solution has been well studied by a spin labeling and CW-EPR spectroscopy approach, which provides information on the maximum number of binding sites per protein as well as the dynamics of SL-pharmaceuticals [39].

CW EPR measurements have proven to be useful for quantitative characterization of drug and fatty acid binding processes to albumin-derived materials and the respective bound states [22,23,50,51]. For albumin hydrogels, results from a recent study on the interaction of 16-DSA with BSA and HSA hydrogels revealed that both proteins and their hydrogels have strong fatty acid binding capacities, however, the number and strength of their binding strongly depends on the gel preparation method [19]. Furthermore, the rotational reorientation as well as polarity around the nitroxide group can be gained from rigorous simulation of EPR spectra. The former, achieved from rotational correlation time *τ_c_,* which is calculated from diffusion tensor *D* (τ_c =_ $\frac{1}{6}{\left(DxxDyyDzz\right)}^{-\frac{1}{3}}$), is in the range of 10 ps for fast rotation components to a few microseconds for tightly immobilized molecules. The latter can be determined from the isotropic ^14^*N*-hyperfine coupling constant α_iso_. High polar environments result in larger α_iso_ values, while smaller hyperfine couplings are indicative of lower polarities [19,20,39]. In the following, we describe the influence of differences in chemical substitution of SL-CCS and SL-WFR on gel formation and their release behavior from BSA hydrogels.

**Figure 3:**
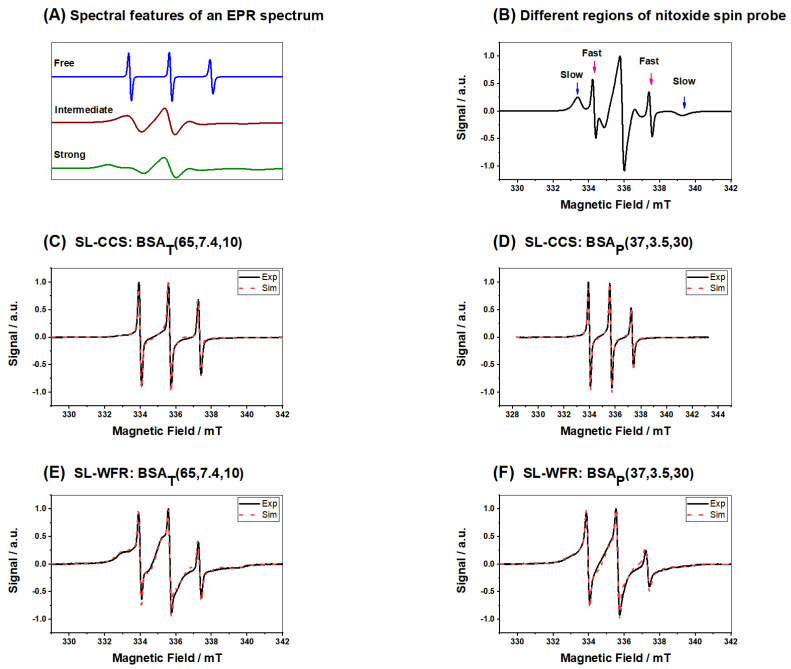
(**A**) Simulated components of EPR spectra; (**B**) different regions of nitroxide spin probe; (**C**) SL-CCS loaded BSA_T_(65, 7.4, 10) at a 2:1 SL-CCS:BSA molar ratio; (**D**) SL-CCS loaded BSA_p_(37, 3.5, 30) at a 2:1 SL-CCS:BSA molar ratio; (**E**) SL-WFR loaded BSA_T_(65, 7.4, 10) at a 2:1 SL-WFR:BSA molar ratio; and (**F**) SL-WFR loaded BSA_p_(37, 3.5, 30) at a 2:1 SL-WFR:BSA molar ratio.

Figure 3A shows the spectral features (simulated) of ligands that are freely tumbling ligands, intermediately, or strongly immobilized in the EPR spectra separately, while in Figure 3B, the features of different spectroscopic species of nitroxide spin probe are displayed in the measured spectrum that essentially is a superposition of these three spectral components of Figure 3A with varying amplitudes (weights). Figure 3C,D shows BSA_T_(65, 7.4, 10) and BSA_P_(37, 3.5, 30) at 2:1 SL-CCS:BSA ratios, respectively. Due to the delayed gel formation of samples prepared by the pH-induced method, much longer incubation times were required. Results from spectral simulation revealed that in a hydrogel prepared by the thermally induced method at 65 °C, almost 26% of SL-CCS showed freely tumbling rotation, 29% were strongly bound to BSA, and 44% had intermediate rotational motion. However, with the addition of acid, the percentage of freely rotating and intermediately immobilized SL-CCS increased to 33% and 51% while that of the strongly bound ligand to BSA decreased to 14%. In general, the strongly immobilized state in BSA could stem from ligand binding sites, which are located at the interfaces of α-helices. The intermediately immobilized ligand with faster rotation is typically situated in water swollen regions of BSA with a β-sheet structure [19]. Acidic conditions affect the helical content in BSA differently to temperature denaturation. In other words, more α-helix content is preserved in pH-induced hydrogels since intermolecular β-sheets formed at the cost of both α-helix and random coils [19]. Figure 3E,F shows the spectra of SL-WFR loaded hydrogels at a 2:1 SL-WFR:BSA molar ratio prepared by heat and pH-change, respectively. Remarkably, the percentage of tightly immobilized and intermediately bound SL-WFR was higher in these hydrogels than in SL-CCS loaded BSA and only less than 10% of SL-WFR rotated freely. The reason for this may be explained by considering the difference in the chemical substitution of both pharmaceuticals (see Figure 1B,C). The free 3-oxo-1-phenyl-butyl group can increase the average binding affinity by providing flexibility to its coumarin backbone in a way that the 3-oxo group can facilitate hydrogen bonding in protein. Compared with SL-WFR, SL-CCS without the additional group has less flexibility and only the carbonyl group at position 2 may have a hydrogen-bond acceptor function. The parameters obtained from simulation of the EPR spectra in Figure 3 are illustrated in Table 1. The results of Figure 3 are comparable to the rheological characterization, as thermally induced hydrogels containing CCS and WFR show more mechanical robustness than the same gels prepared through reducing pH. Moreover, as will be explained later, there were high percentages of freely tumbling SL-pharmaceuticals in the release medium of samples prepared by the thermally induced method, and there was no sign of strongly bound components. Therefore, we can conclude that the SL-pharmaceuticals are also bound more strongly to these types of hydrogels. The hydrogel characterization for both SL-pharmaceuticals at other ratios and temperatures is described in more detail in the Supplementary Materials.

**Figure 4:**
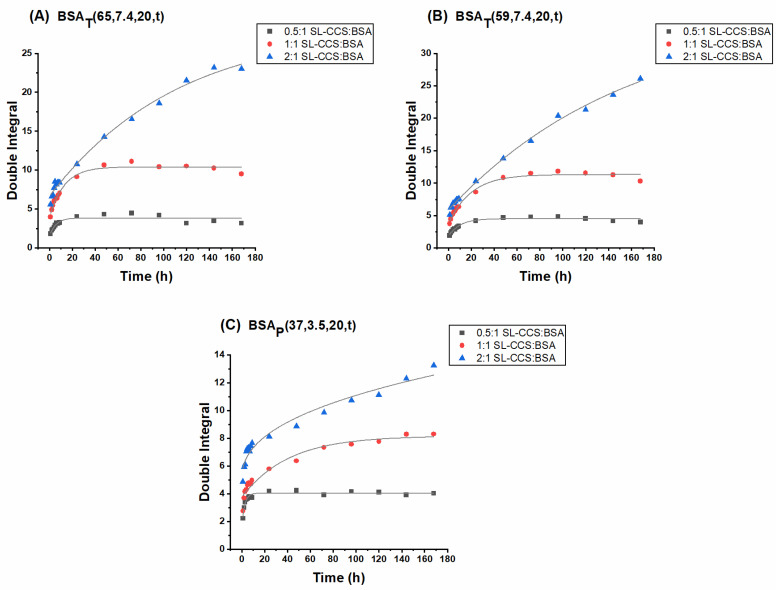
Release profiles of SL-CCS loaded BSA hydrogels at 0.5:1, 1:1 and 2:1 SL-CCS:BSA molar ratios: (**A**) BSA_T_(65, 7.4, 20) hydrogel; (**B**) BSA_T_(59, 7.4, 20) hydrogel; and (**C**) BSA_P_(37, 3.5, 20) hydrogel. The curves are fitted.

**Figure 5:**
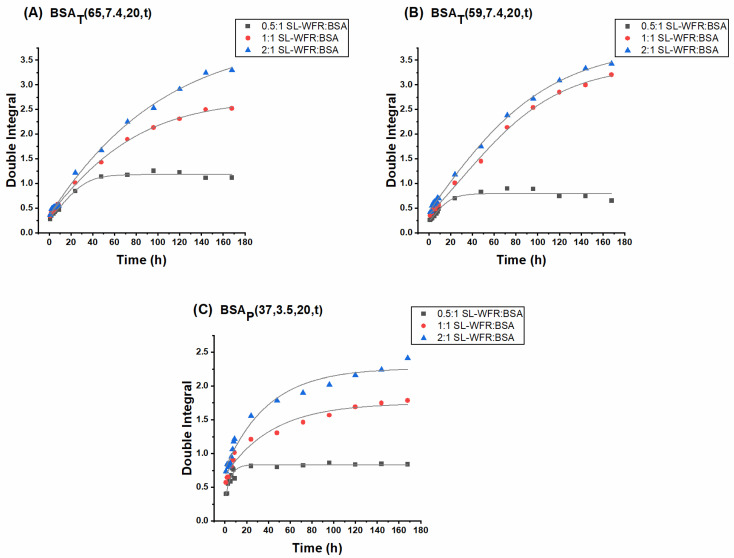
Release profiles of SL-WFR loaded BSA hydrogels with 0.5:1, 1:1 and 2:1 SL-CCS:BSA molar ratios: (**A**) BSA_T_(65, 7.4, 20) hydrogel; (**B**) BSA_T_(59, 7.4, 20) hydrogel; and (**C**) BSA_P_(37, 3.5, 20) hydrogel. The curves are fitted.

In Figure 4 and Figure 5, the release profiles of SL-CCS and SL-WFR from BSA hydrogels are given at different ratios and by the two preparation methods (other release curves for hydrogels with lower incubation times can be found in the Supplementary Materials). Double integration of the first derivative CW EPR spectra can be used to determine the signal intensity of the spectra [52]. Therefore, in all release curves, the double integral of EPR spectra is plotted versus the release time intervals.

It can be seen that all 0.5:1 and 1:1 ratios in the SL-CCS loaded BSA hydrogels prepared by different methods showed an initial fast release before reaching a plateau after 24 h. The initial release of large amounts of drug corresponds with the diffusion of release medium into the protein network, which dissolves the entrapped SL-CCS close to or at the surface of the hydrogel. However, the second and slower release is due to the depletion of drug in the inner part of gel matrix, which leads to the increase in the diffusion process length. In contrast, the kinetics of the SL-CCS:BSA 2:1 ratios almost corresponded to a zero-order release with an initial burst effect. It is possible that by increasing the ratio to 2:1, the binding sites from which the later release occurred are fully occupied and instead, the amount of drug between the hydrogel networks increases, which results in a drug release at a constant rate.

Figure 5 summarizes the SL-WFR release data. One can see an initial burst release for all samples, and a later sustained release only at the 0.5:1 SL-WFR:BSA molar ratio. The release from gels at 1:1 and 2:1 SL-WFR:BSA molar ratios first clearly followed the zero order kinetics and flattened after ~100h. Since BSA has only one high affinity binding site for SL-WFR, increasing the ratio to 1:1 and 2:1 resulted in the presence of a greater amount of freely moving SL-drug on the hydrogel surface and between the protein networks, which led to the linear release during the first five days; afterward, the release reached a certain level at which there was no more free SL-WFR available and the release of those from the binding site is initiated. We observed that all hydrogels with SL-CCS, regardless of molar ratios and preparation methods, had a higher release rate than SL-WFR loaded BSA gels, which may be attributed to the higher affinity of SL-WFR to albumin compared to SL-CCS. Therefore, SL-WFR tends to attach to the binding pockets of BSA for longer periods of time and shows a lower release rate.

The effect of initial drug loading and preparation methods on the release profile is also discussed in Figure 4 and Figure 5. In all experimental systems, we found that the increase in the ratio of SL-pharmaceuticals to albumin led to a higher release rate. In addition, thermally induced hydrogels prepared at 65 °C and 59 °C, respectively, had higher release rates than pH-induced hydrogels. As explained above for the rheological characterization, electrostatically triggered hydrogels are less rigid compared to hydrogels formed by heating, which may lead to the assumption that these gels release their pharmaceuticals faster. However, we observed the opposite effect. This is possibly because there are stronger transient interactions/attachments between the pharmaceuticals and the increased protein surface under acidic conditions. These transient complexes could protect the hydrogel from fast degradation, which may lead to a slower release rate over time. However, when comparing the release rate of hydrogels prepared at 65 °C with the respective rate for the gel prepared at 59 °C, the results showed that hydrogels that formed below the BSA denaturation temperature (at 59 °C) had a slightly higher rate of release as these gels were mechanically less robust than those prepared at 65 °C.

**Figure 6:**
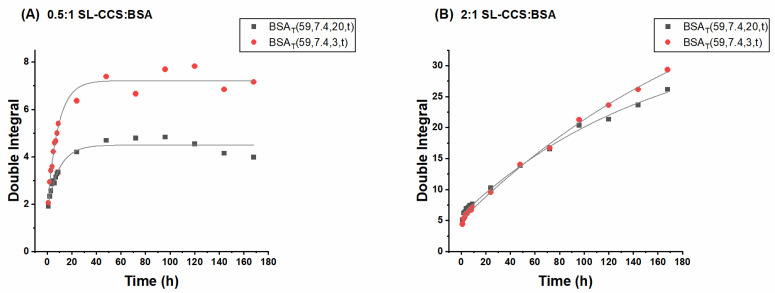
Release profiles of SL-CCS loaded BSA hydrogels prepared using heat induction at 59 °C with two different incubation times of 3 and 20 min at a (**A**) 0.5:1 SL-CCS:BSA molar ratio and (**B**) 2:1 SL-CCS:BSA molar ratio. The curves are fitted.

Figure 6 shows the effect of incubation time on the release behavior of BSA hydrogels incorporated with different ratios of SL-CCS (other ratios of SL-CCS and SL-WFR loaded hydrogels are available in the Supplementary Materials). It seems that increasing the incubation time from 3 min to 20 min slightly decreased the release rate. These results are in line with those of the rheological characterization, as a longer duration of gelation leads to more mechanically robust hydrogels. Consequently, the release rate can be tuned by changing the incubation time, which indicates the remarkable potential of BSA hydrogels for their implementation in controlled drug delivery applications.

**Figure 7:**
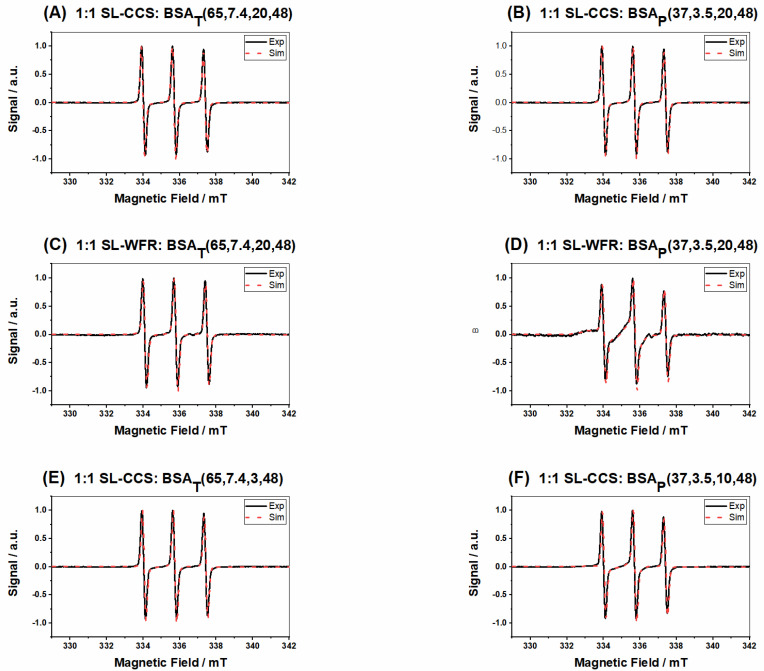
EPR spectra of release test after 48 h for SL-drugs loaded BSA hydrogels at a 1:1 molar ratio (**A**) SL-CCS:BSA_T_(65, 7.4, 20, 48), (**B**) SL-CCS:BSA_p_(37, 3.5, 20, 48), (**C**) SL-WFR:BSA_T_(65, 7.4, 20, 48), (**D**) SL-WFR: BSA_p_(37, 3.5, 20, 48), (**E**) SL-CCS:BSA_T_(65, 7.4, 3, 48), and (**F**) SL-CCS:BSA_P_(37, 3.5, 10, 48).

Figure 7 displays EPR spectra gained by analyzing the release medium. More molecular insights were obtained by spectral simulation, providing information on the fraction of SL-drugs strongly and intermediately bound to BSA and freely rotating SL-pharmaceuticals in the released solution (see Table 2). Results showed that 84% of SL-CCS rotated freely in PBS, 16% showed intermediate rotational motion, and there was no sign of tightly bound SL-CCS to BSA for the sample prepared by the heat-induced method at 65 °C by keeping the sample in a thermomixer for 20 min (the EPR spectra of the respective release experiment for samples prepared at 59 °C are available in the Supplementary Materials). The BSA hydrogel with SL-WFR prepared at the same temperature and incubation time showed an, in principal, similar release behavior. However, the percentage of freely tumbling SL-WFR decreased to 73% and that of the intermediate component increased to 26%. The percentage of strongly bound SL-drugs to BSA in the release medium increased when the hydrogels were prepared by the pH-induced method at 37 °C. For instance, 19% of SL-CCS and 46% of SL-WFR were released as they bind tightly to BSA. It must be mentioned that the percentage of released components in bound, intermediate, and free states was nearly constant during the different release time intervals for all samples, while in our previous work on fatty acid release, these percentages changed during the release experiment [20].

EPR spectra of the released components from BSA hydrogels with SL-CCS prepared with lower incubation times (3 min for the thermally induced hydrogel at 65 °C and 10 min for the pH-induced gel at 37 °C) are shown in Figure 7E,F. According to Table 2, we found that reducing the incubation time led to the release of a more intermediately immobilized SL-drug to BSA in the heat-induced gel and intermediately and strongly bound SL-CCS to BSA for the electrostatically triggered gel (see the Supplementary Materials the for release from hydrogels made at 59 °C and the whole data of SL-WFR incorporated BSA gels).

### 3.3. Monitoring of Release Behavior by Means of EPR Imaging

We applied electron paramagnetic resonance imaging (EPRI) to study the spatial localization of a spin probe in a nondestructive way and followed the position of the probes inside the slowly dissolving gels not in the release medium. This technique combines spectral information with the spatial distribution of paramagnetic species, which permits monitoring of the changes in the property in different parts of the investigated sample [52,53].

**Figure 8:**
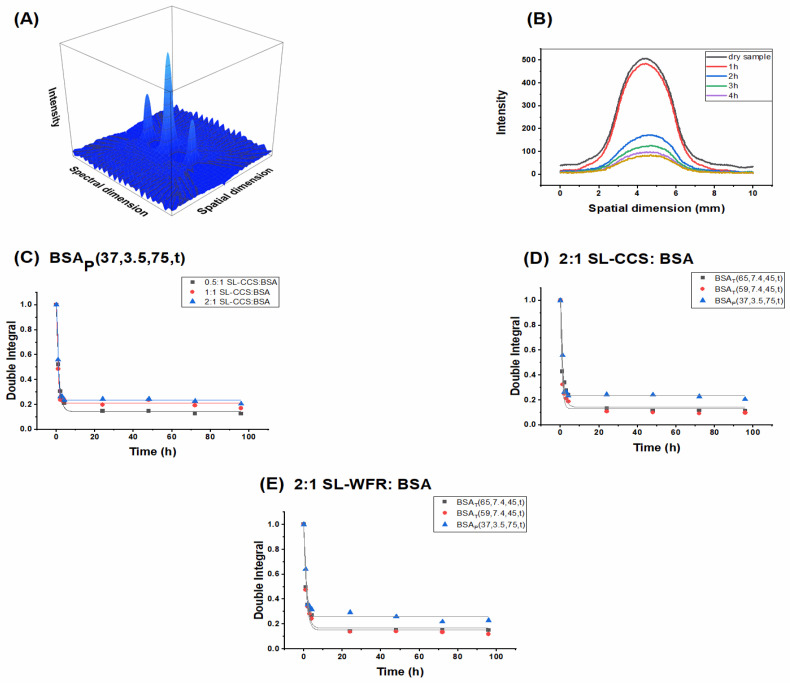
(**A**) 2-D spectral-spatial image of the BSA hydrogel with SL-CCS at a spin probe:BSA 1:1 molar ratio prepared by the heat induced method at 65 °C. (**B**) Central spatial cut (a) top and (b) bottom of the dry SL-CCS loaded BSA_T_(65, 7.4, 45) and release of SL-CCS from the BSA hydrogel during the first 4 h. Double integration of central spatial cuts of (**C**) SL-CCS:BSA_P_(37, 3.5, 75, t) at different molar ratios, (**D**) 2:1 SL-CCS:BSA molar ratio prepared with heat and pH induced methods, and (**E**) 2:1 SL-WFR:BSA molar ratio prepared with heat and pH induced methods. The curves are fitted.

Figure 8A shows a spectral-spatial two dimensional EPR image (absorption mode, not the standard CW EPR first-derivative mode) of a SL-CCS loaded BSA hydrogel. The intensity and spectral dimensions of a typical EPR spectrum are plotted along the z- and *y*-axes, respectively; the spatial dimension is shown along the *x*-axis. By applying spatial cuts, it is possible to obtain information on intensity distributions at certain positions. Figure 8B depicts the central spatial cut of dry SL-CCS loaded BSA_T_(65, 7.4, 3) and release of SL-CCS from the BSA hydrogel during the first 4 h. It can be seen that the signal amplitude decreased over time, which illustrates the release of the SL-pharmaceutical from the hydrogel. Moreover, the EPR signal intensities slightly shifted to the right (bottom of the hydrogel) with release time, indicating that release occurs from the gel surface.

The amount of SL-pharmaceuticals inside the BSA hydrogel was calculated by double integration of the central spatial cuts. Figure 8C displays the amount of SL-CCS inside the BSA hydrogels prepared by the pH-induced method after exposure to the PBS buffer. For a better comparison, all of the samples were normalized by dividing each data point by the highest double integral value, the dry samples, for each of the ratios. We can see that the hydrogel at a 0.5:1 SL-CCS:BSA had the lowest amount of spin probe inside the gel due to the lowest initial drug content, while increasing the drug loading percentage led to a higher drug quantity in hydrogels over the whole release period. Figure 8D,E shows the effect of preparation methods on the release profile of SL-CCS and SL-WFR from BSA hydrogels. According to the double integral values, heat induced gels contained lower amount of drugs, while pH-induced hydrogels preserved a higher quantity of drug within their networks. These results are in agreement with the CW-EPR measurements, as hydrogels thermally induced at 65 °C and 59 °C showed higher release rates in comparison to the electrostatically triggered hydrogels. As explained in the CW-EPR section, acidic conditions may induce more interactions between pharmaceuticals and the protein surface, which results in a higher protein protection from fast degradation and lower release rate over time. Furthermore, all hydrogels with different ratios of SL-CCS or SL-WFR prepared with different methods showed that they released almost 80% of their drug contents during the first 24 h. The sustained release phase in all experimental samples is thought to involve the penetration of PBS into the protein networks, leading to the final dissolution of the drug in the inner part of the BSA chains. However, the results from CW-EPR showed zero-order kinetics for some ratios. It is possible that the difference in hydrogel quantities, sample holders used for CW-EPR and EPRI, or incubation times needed for gel formation leads to different release patterns.

### 3.4. Analyzing the Released Components with DLS

**Figure 9:**
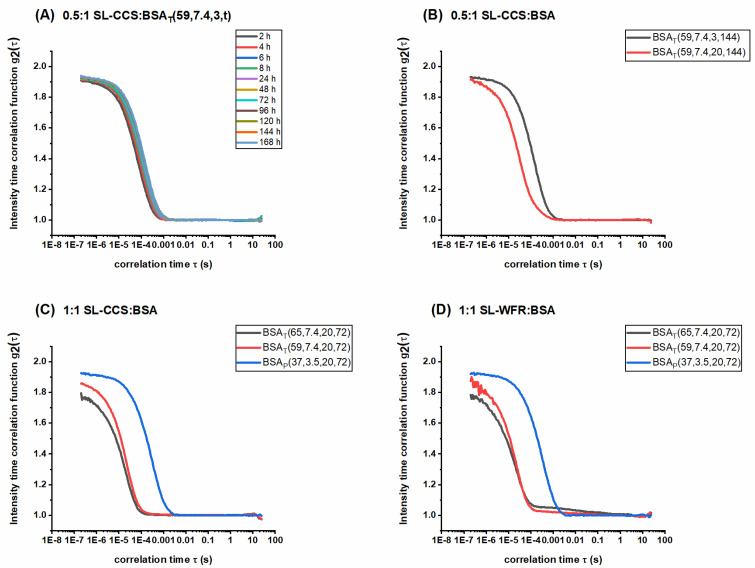
Intensity time correlation functions for (**A**) 0.5:1 SL-CCS:BSA_T_(59, 7.4, 3) molar ratio during different release times, (**B**) SL-CCS:BSA_T_(59, 7.4, 3, 144) and SL-CCS:BSA_T_(59, 7.4, 20, 144) at a 0.5:1 molar ratio, (**C**) SL-CCS:BSA_T_(65, 7.4, 20, 72), SL-CCS:BSA_T_(59, 7.4, 20, 72) and SL-CCS:BSA_P_(37, 3.5, 20, 72) at a 1:1 molar ratio, and (**D**) SL-WFR:BSA_T_(65, 7.4, 20, 72), SL-WFR:BSA_T_(59, 7.4, 20, 72), and SL-WFR: BSA_P_(37, 3.5, 20, 72) at a 1:1 molar ratio.

To better understand the components released over time, we determined the hydrodynamic radii and correlation functions using dynamic light scattering (DLS). Figure 9 displays the intensity–time correlation function of the released medium. We recorded pronounced autocorrelation functions and high scattering intensities, implying the presence of highly defined particles in PBS buffer. Figure 9A shows the autocorrelation functions from different release times from the heat induced SL-CCS incorporated BSA hydrogel prepared at 59 °C. We found that all autocorrelation functions decayed almost at the same time, indicating the release of at least one particle with the same size over release time periods. Figure 9B shows the effect of changing the incubation time on the size of the released components for hydrogels made with the same concentration, preparation method, and release time. It can clearly be seen that a sample with higher incubation time (20 min) decayed faster, which correlates with the release of smaller particles. These results are in good agreement with the rheological characterization and CW-EPR spectroscopy as hydrogels prepared with lower incubation times are mechanically less robust, which may result in the release of larger albumin derivatives formed during the gelation process. Furthermore, results from the simulation of the EPR spectra showed slightly higher percentages of SL-CCS and SL-WFR intermediately bound to BSA released from the hydrogels with lower incubation times (see Supplementary Materials). Further information about the release of SL-WFR from BSA hydrogels with different incubation times and behavior of the hydrodynamic radius can be found in the Supplementary Materials.

Figure 9C,D shows the autocorrelation functions of SL-CCS and SL-WFR release from the BSA hydrogels after 72 h and the gels prepared with both thermally and pH induced methods by keeping the samples in the thermomixer for 20 min (results on the lower incubation time of SL-drug incorporated BSA hydrogels are given in the Supplementary Materials). As can be seen in both figures, the autocorrelation functions of the electrostatically triggered hydrogels displayed a slower decay those of the hydrogels prepared by the heat induced method at 65 °C and 59 °C. As discussed in the rheology section, the addition of acid to a precursor solution of BSA led to a mechanically weak hydrogel. Moreover, according to Table 2, the percentage of SL-pharmaceuticals strongly immobilized to BSA in the release medium increased when the hydrogels were prepared by the pH-induced method. We observed the same behavior in the DLS measurements. The existence of strongly bound SL-drugs, which are larger structures than intermediately and freely tumbling ones, results in the slower decay of the autocorrelation function. However, if—like in thermally-induced hydrogels—only freely rotating and intermediately BSA-bound SL-pharmaceuticals are released from thermally induced hydrogels, leading to the fast decay of the autocorrelation function for these samples. Therefore, these results complement all the earlier results by adding the perspective on the (larger) structures that are actually released into the release medium.

## 4. Conclusions

In the present work, BSA hydrogels with good mechanical properties were prepared as delivery hosts for pharmaceutical applications. The whole gelation procedures, namely thermally and pH induced methods, have previously been explained in detail [19]. SL-CCS and SL-WFR were loaded into BSA hydrogels at different molar ratios to evaluate the applicability of these structures as drug delivery systems. The results suggest that the drug release rate gained from double integration of EPR spectra is dependent on the type of drug, drug ratios, incubation time, and gelation method. We found that SL-CCS had a much higher release rate than SL-WFR. In general, higher drug concentrations, lower incubation times, and heat induced hydrogels prepared below BSA denaturation temperature resulted in a higher rate of release. Furthermore, the drug release pattern from these hydrogels showed that they can be used where the fast onset of release is required. The drug loading can affect the release behavior as lower ratios indicated the second sustained release phase, while higher ratios showed zero-order kinetics. Analyzing the hydrolytic degradation of hydrogels in the presence and absence of drugs as well as the SEM images of gels will be part of future work.

Additionally, EPR spectroscopy and DLS were applied to shed more light onto the interaction of SL-pharmaceuticals with BSA hydrogels as well as the nature and size of the released structures. We have shown that differences in chemical substitutions of SL-CCS and SL-WFR and binding capacities of BSA for SL-pharmaceuticals can deeply affect drug–protein interactions. Since a higher percentage of SL-WFR is attached strongly and intermediately to BSA hydrogels, more of these components are released compared to the SL-CCS incorporated BSA hydrogel, in which a higher percentage of freely tumbling SL-drug was found in the release medium. Moreover, a higher percentage of SL-CCS/SL-WFR intermediately (in case of thermally and pH induced hydrogels) and strongly (for electrostatically-triggered gels) bound to BSA was released from less rigid hydrogels due to faster penetration of water into the gel network. This led to the expedited release of albumin dimers or individual proteins when compared to the mechanically robust hydrogels. We hope that this study aids in paving the way to employing the full potential of BSA hydrogels as a suitable controlled release system and their implementation in biomedical applications.

## Figures and Tables

**Table 1:** Parameters gained from the simulation in Figure 3.

Figure	Type of Released Components	Percentage	Correlation Time *τ*c (ns)	Hyperfine Coupling Constant a_iso_ (MHz)
C	Bound	29%	14.6	44.53
	Intermediate	44%	5.4	47.53
	Free	26%	0.11	46.86
D	Bound	10%	14.5	44.53
	Intermediate	54%	4.76	47.53
	Free	35%	0.16	46.86
E	Bound	47%	31	42.53
	Intermediate	48%	3.2	46.86
	Free	3%	0.061	47.13
F	Bound	36%	31	42.53
	Intermediate	56%	3.2	46.86
	Free	6%	0.85	47.20

**Table 2:** Percentage of released components in different states gained from the spectral simulation in Figure 7.

Figure	Type of Released Components	Percentage (Bound, Intermediate and Free)	Figure	Type of Released Components	Percentage (Bound, Intermediate and Free)
A	Bound	0	D	Bound	46%
	Intermediate	16%		Intermediate	31%
	Free	84%		Free	22%
B	Bound	19%	E	Bound	0
	Intermediate	16%		Intermediate	20%
	Free	63%		Free	80%
C	Bound	0	F	Bound	24%
	Intermediate	26%		Intermediate	19%
	Free	73%		Free	55%

## Data Availability

Data are available from the authors upon request.

## Supplementary Materials

The following are available online at https://www.mdpi.com/article/10.3390/pharmaceutics13101661/s1, Figure S1: Storage (G′) and loss (G″) moduli as a function of time for different ratios of CCS:BSA at 59 °C, pH 7.4, Figure S2: Storage (G′) and loss (G″) moduli as a function of time for different ratios of WFR:BSA at (A) 65 °C, pH 7.4, (B) 59 °C, pH 7.4 and (C) 37 °C, pH 3.5, Figure S3: SL-CCS loaded (A) BSAT(65, 7.4, 10) at a 0.5:1 SL-CCS:BSA molar ratio, (B) BSAT(65, 7.4, 10) at a 1:1 SL-CCS:BSA molar ratio, (C) BSAT(59, 7.4, 10) at a 0.5:1 SL-CCS:BSA molar ratio, (D) BSAT(59, 7.4, 10) at a 1:1 SL-CCS:BSA molar ratio, (E) BSAT(59, 7.4, 10) at a 2:1 SL-CCS:BSA molar ratio, (F) BSAP(37, 3.5, 30) at a 0.5:1 SL-CCS:BSA molar ratio and (G) BSAP(37, 3.5, 30) at a 1:1 SL-CCS:BSA molar ratio, Figure S4: SL-WFR loaded (A) BSAT(65, 7.4, 10) at a 0.5:1 SL-WFR:BSA molar ratio, (B) BSAT(65, 7.4, 10) at a 1:1 SL-WFR:BSA molar ratio, (C) BSAT(59, 7.4, 10) at a 0.5:1 SL-WFR:BSA molar ratio, (D) BSAT(59, 7.4, 10) at a 1:1 SL-WFR:BSA molar ratio, (E) BSAT(59, 7.4, 10) at a 2:1 SL-WFR:BSA molar ratio, (F) BSAP(37, 3.5, 30) at a 0.5:1 SL-WFR:BSA molar ratio and (G) BSAP(37, 3.5, 30) at a 1:1 SL-WFR:BSA molar ratio, Figure S5: Release profiles of SL-CCS loaded BSA hydrogels with 0.5:1, 1:1 and 2:1 SL-CCS:BSA molar ratios (A) BSAT(65, 7.4, 3) hydrogel, (B) BSAT(59, 7.4, 3) hydrogel and (C) BSAP(37, 3.5, 10) hydrogel. The curves are fitted, Figure S6: Release profiles of SL-WFR loaded BSA hydrogels with 0.5:1, 1:1 and 2:1 SL-WFR:BSA molar ratios (A) BSAT(65, 7.4, 3) hydrogel, (B) BSAT(59, 7.4, 3) hydrogel and (C) BSAP(37, 3.5, 10) hydrogel. The curves are fitted, Figure S7: Release profiles of SL-CCS and SL-WFR loaded BSA hydrogels prepared with heat induced method at 59 °C with two different incubation times of 3 and 20 min at a (A) 0.5:1 SL-CCS:BSA molar ratio, (B) 0.5:1 SL-WFR:BSA molar ratio, (C) 1:1 SL-WFR:BSA molar ratio and (D) 2:1 SL-WFR:BSA molar ratio, Figure S8: EPR spectra of release test after 48 h for SL-drugs loaded BSA hydrogels at a 1:1 molar ratio (A) SL-CCS:BSAT(59, 7.4, 20, 48) and (B) SL-WFR:BSAT(59, 7.4, 20, 48), Figure S9: EPR spectra of release test after 48 h for SL-drugs loaded BSA hydrogels at a 1:1 molar ratio (A) SL-CCS:BSAT(59, 7.4, 3, 48), (B) SL-WFR:BSAT(65, 7.4, 3, 48), (C) SL-WFR:BSAT(59, 7.4, 3, 48) and (D) SL-WFR:BSAT(37, 3.5, 10, 48), Figure S10: Intensity time correlation functions for 0.5:1 SL-WFR:BSAT(59, 7.4, 3) molar ratio during different release times, Figure S11: Intensity time correlation functions for different concentrations of (A) SL-CCS diluted with 10× PBS and (B) SL-WFR diluted with 10× PBS, Figure S12: (A) Intensity time correlation functions for different concentrations of BSA solution diluted by 10× PBS and (B) hydrodynamic radius of different concentrations of BSA solution diluted by 10× PBS, Figure S13: Intensity time correlation functions of (A) SL-CCS:BSAT(59, 7.4, 3, 144) and SL-CCS:BSAT(59, 7.4, 20, 144) at a 1:1 molar ratio and (B) SL-CCS:BSAT(59, 7.4, 3, 144) and SL-CCS:BSAT(59, 7.4, 20, 144) at a 2:1 molar ratio, Figure S14: Intensity time correlation functions of (A) SL-WFR:BSAT(59, 7.4, 3, 144) and SL-WFR:BSAT(59, 7.4, 20, 144) at a 0.5:1 molar ratio, (B) SL-WFR:BSAT(59, 7.4, 3, 144) and SL-WFR:BSAT(59, 7.4, 20, 144) at a 1:1 molar ratio and (C) SL-WFR:BSAT(59, 7.4, 3, 144) and SL-WFR:BSAT(59, 7.4, 20, 144) at a 2:1 molar ratio, Figure S15: Intensity time correlation functions for (A) SL-CCS:BSAT(65, 7.4, 3, 72), SL-CCS:BSAT(59, 7.4, 3, 72) and SL-CCS:BSAP(37, 3.5, 10, 72) at a 1:1 molar ratio and (B) SL-WFR:BSAT(65, 7.4, 3, 72), SL-WFR:BSAT(59, 7.4, 3, 72) and SL-WFR:BSAP(37, 3.5, 10, 72) at a 1:1 molar ratio, Table S1: Parameters gained from simulation of Figure S3, Table S2: Parameters gained from simulation of Figure S4, Table S3: Percentage of released components in different states gained from spectral simulation of Figure S8, Table S4: Percentage of released components in different states gained from spectral simulation of Figure S9.
