# Supplementary material for: Molecular-Level Release of Coumarin-3-Carboxylic Acid and Warfarin-Derivatives from BSA-Based Hydrogels
Source: Pharmaceutics. 2021 Oct 11;13(10):1661. doi: 10.3390/pharmaceutics13101661 (PMC8539358; doi:10.3390/pharmaceutics13101661)
Supplement: Supplementary file 1 [file pharmaceutics-13-01661-s001.zip › pharmaceutics-1384546-si.pdf]

pharmaceutics

MDPI

Supplementary Materials: Molecular-Level Release of Coumarin-3-Carboxylic Acid and Warfarin-Derivatives from BSA-Based Hydrogels

Niuosha Sanaeifar, Karsten Mäder and Dariush Hinderberger

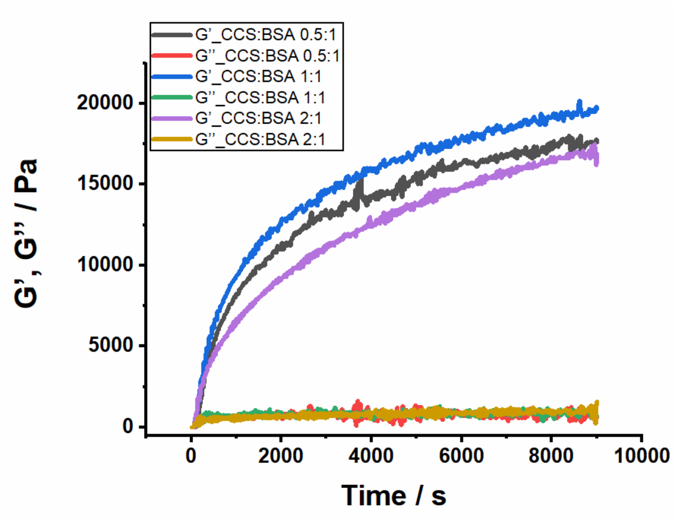

Figure S1. Storage (G′) and loss (G″) moduli as a function of time for different ratios of CCS:BSA at 59 °C, pH 7.4.

Figure S1 demonstrates the viscoelastic behavior of the BSA precursor solution with different ratios of CCS at 59 °C. It shows the same pattern and behavior as the samples prepared at 65 °C. The addition of CCS reduces the storage modulus and gel strength in comparison with the hydrogel formed at 59 °C without any CCS. There is no considerable difference in the storage modulus of hydrogels with different ratios of CCS. We can conclude that CCS concentration did not play a significant role on mechanical properties.

Time dependence of storage and loss moduli of different ratios of WFR: BSA solution at different temperatures are depicted in Figure S2. We can see the same behavior as CCS was added to the precursor solution of BSA. Thermally induced hydrogels (at 65 °C and 59 °C) with different ratios of WFR are less rigid than the one formed with BSA alone. Due to the Sudlow binding sites in albumin which have affinities for various drugs, conformational changes are impeded and BSA molecule is more stabilized. Moreover, different ratios of WFR did not affect the gelation and the storage moduli after the addition of different WFR concentrations remain almost the same. However, the opposite behavior can be seen in the samples prepared by pH induced method. The addition of WFR until WFR: BSA 1:1 molar ratio speed up the gelation process and leads to slightly higher G′ value compared to the gel without WFR (see Figure 1 in main manuscript). Although, it seems that by increasing the ratio to WFR: BSA 2:1 the storage modulus decreases.

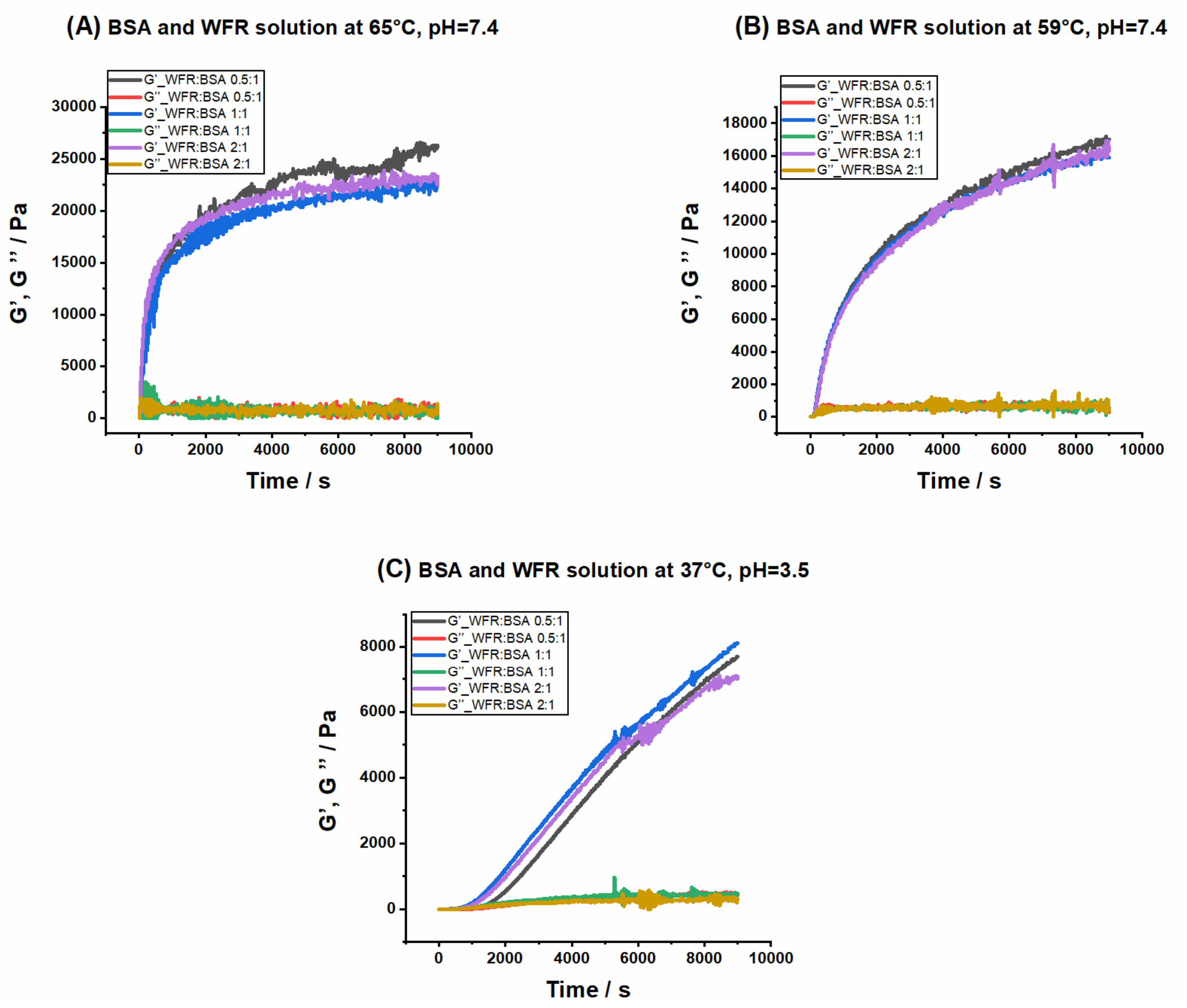

Figure S2. Storage (G′) and loss (G″) moduli as a function of time for different ratios of WFR: BSA at (**A**) 65 °C, pH 7.4, (**B**) 59 °C, pH 7.4 and (**C**) 37 °C, pH 3.5.

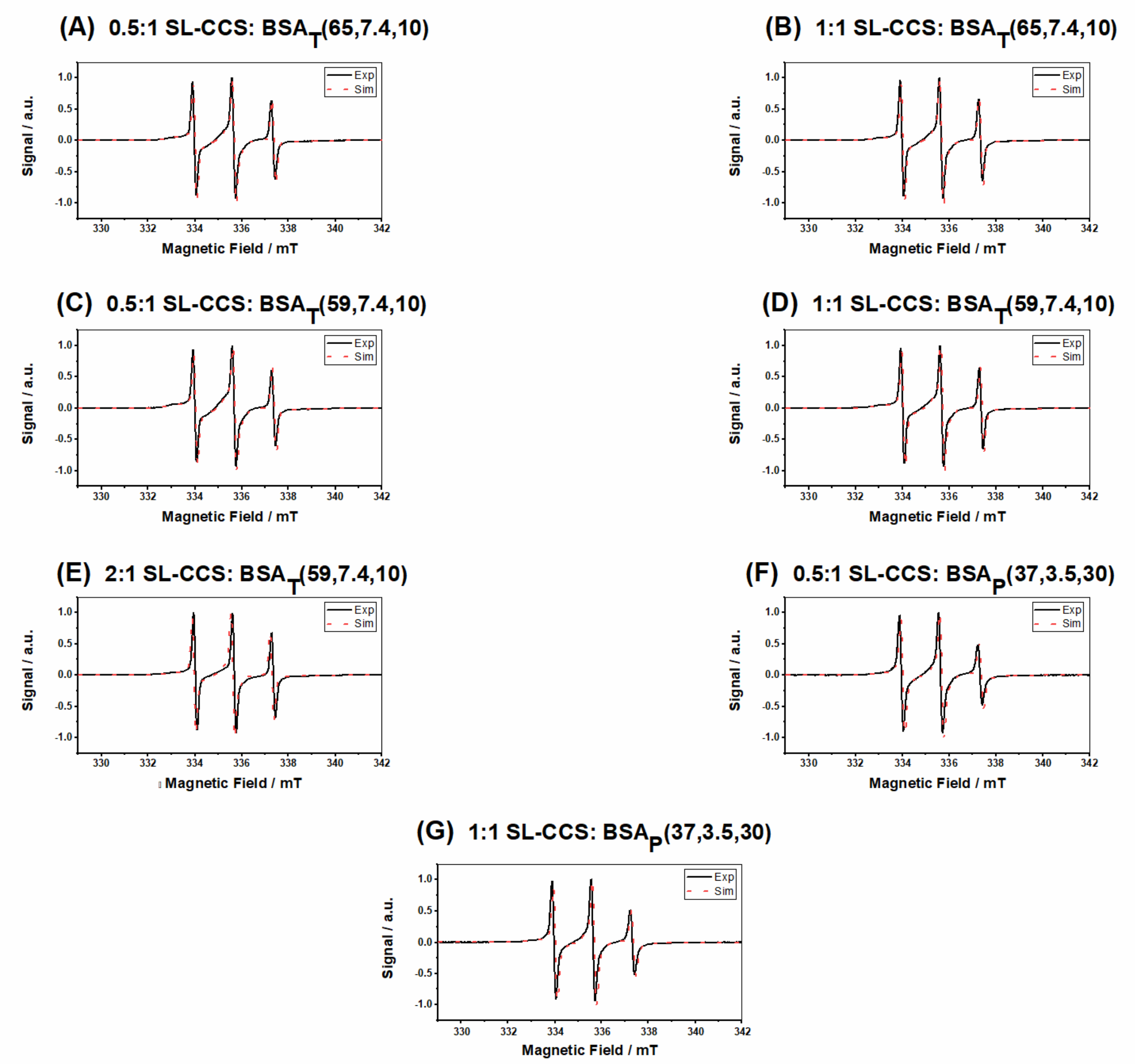

Figure S3. SL-CCS loaded (**A**) BSA_T(65, 7.4, 10) at a 0.5:1 SL-CCS: BSA molar ratio, (**B**) BSA_T(65, 7.4, 10) at a 1:1 SL-CCS: BSA molar ratio, (**C**) BSA_T(59, 7.4, 10) at a 0.5:1 SL-CCS: BSA molar ratio, (**D**) BSA_T(59, 7.4, 10) at a 1:1 SL-CCS: BSA molar ratio, (**E**) BSA_T(59, 7.4, 10) at a 2:1 SL-CCS: BSA molar ratio, (**F**) BSA_P(37, 3.5, 30) at a 0.5:1 SL-CCS: BSA molar ratio and (**G**) BSA_P(37, 3.5, 30) at a 1:1 SL-CCS: BSA molar ratio.

Table S1. Parameters gained from simulation of Figure S3.

Figure	Percentage	Correlation time τc [ns]	Hyperfine coupling constant a_{iso} [MHz]
**A**	29%	14	44.73
	49%	5.06	47.53
	21%	0.11	47.36
**B**	27%	14	44.73
	49%	5.06	47.53
	23%	0.11	47.03
**C**	35%	14	44.73
	46%	5.06	47.53
	17%	0.11	47.26
**D**	31%	14	44.73
	47%	5.06	47.53
	20%	0.11	47.20
**E**	31%	14	44.53
	44%	5.06	47.53
	23%	0.11	46.86
**F**	21%	14	44.53
	51%	4.7	47.53
	27%	0.16	47.20
**G**	22%	14	44.53
	47%	4.7	47.53
	29%	0.16	47.20

Figure S3 shows the simulated EPR spectra of SL-CCS loaded hydrogels prepared by heat and pH induced methods at different SL-CCS: BSA molar ratios. According to Table S1 which depicts the parameters obtained from rigorous simulation of Figure S3, hydrogels prepared at 59 °C give larger amount of immobilized SL-CCS to BSA compared to ones prepared above denaturation temperature at 65 °C. Moreover, as explained in the main manuscript acidic condition can affect interaction of SL-pharmaceutical with BSA, since by addition of HCl the percentage of bound component decreases and that of the intermediately and freely tumbling SL-CCS increases.

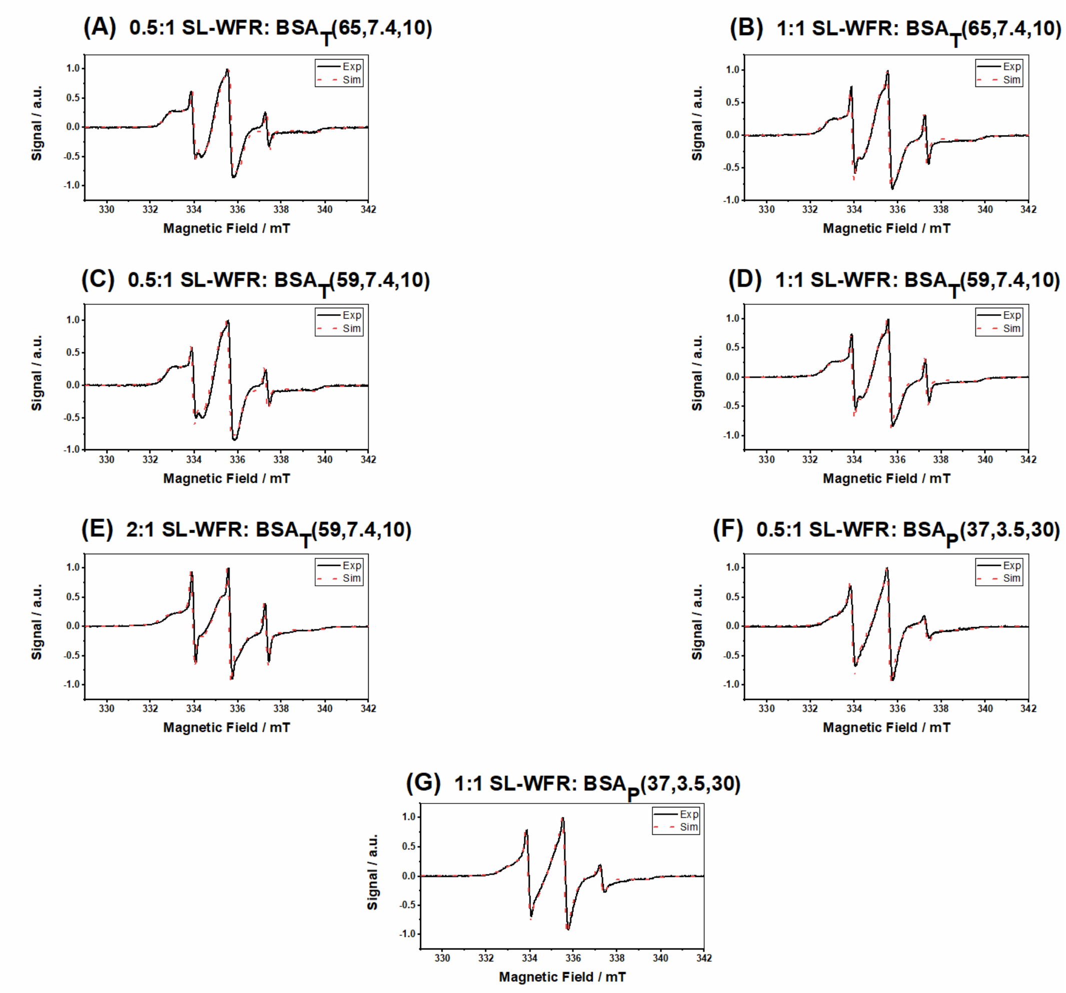

Figure S4. SL-WFR loaded (**A**) BSA$_T$(65, 7.4, 10) at a 0.5:1 SL-WFR:BSA molar ratio, (**B**) BSA$_T$(65, 7.4, 10) at a 1:1 SL-WFR:BSA molar ratio, (**C**) BSA$_T$(59, 7.4, 10) at a 0.5:1 SL-WFR:BSA molar ratio, (**D**) BSA$_T$(59, 7.4, 10) at a 1:1 SL-WFR:BSA molar ratio, (**E**) BSA$_T$(59, 7.4, 10) at a 2:1 SL-WFR:BSA molar ratio, (**F**) BSA$_P$(37, 3.5, 30) at a 0.5:1 SL-WFR:BSA molar ratio and (**G**) BSA$_P$(37, 3.5, 30) at a 1:1 SL-WFR:BSA molar ratio.

Table S2. Parameters gained from simulation of Figure S4.

Figure	Percentage	Correlation time τc (ns)	Hyperfine coupling constant a_{iso} (MHz)
**A**	42%	32	44.73
	55%	7.01	46.53
	2%	0.061	47.60
**B**	43%	25	44.8
	54%	6.3	46.53
	3%	0.06	47.26
**C**	42%	32	44.73
	55%	7.1	46.53
	2%	0.06	47.26
**D**	52%	25	44.86
	43%	5.9	46.53
	3%	0.061	47.26
**E**	47%	30	42.53
	48%	2.1	45.20
	3%	0.06	47.13
**F**	41%	23	44.1
	49%	6.5	47.53
	8%	0.17	47.20
**G**	41%	23	44.53
	49%	7.05	46.53
	8%	0.17	47.20

Figure S4 shows the EPR spectra of SL-WFR loaded BSA hydrogels prepared with two preparation techniques at different ratios of SL-WFR. By comparing the results obtained from simulation of hydrogels incorporated with SL-WFR (Table S2) with those with SL-CCS, we can see that higher percentage of components are in bound and intermediate states, while only a small fraction of SL-WFR rotates freely. This is due to the difference in chemical substitution of both pharmaceuticals. The side chain of SL-WFR which provides flexibility to its coumarin backbone and the 3-oxo group has hydrogen-bond acceptor function can lead to higher protein affinities.

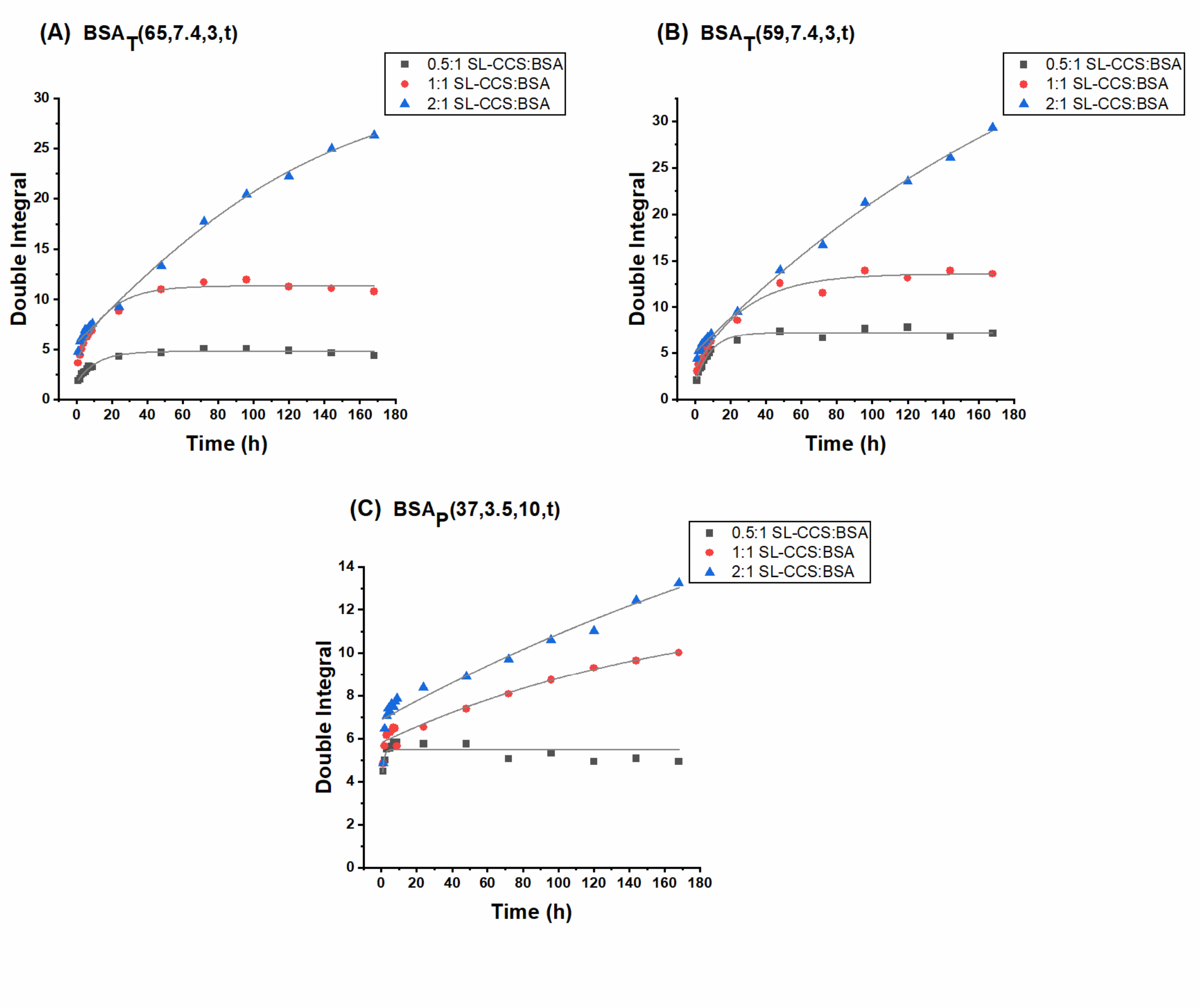

Figure S5. Release profiles of SL-CCS loaded BSA hydrogels with 0.5:1, 1:1 and 2:1 SL-CCS: BSA molar ratios (**A**) BSA$_T$(65, 7.4, 3) hydrogel, (**B**) BSA$_T$(59, 7.4, 3) hydrogel and (**C**) BSA$_P$(37, 3.5, 10) hydrogel. The curves are fitted.

Figure S5 displays SL-CCS release profiles from BSA hydrogels prepared by thermally and pH induced methods with lower incubation times. The release behavior is identical to hydrogels prepared with higher incubation times (see the main manuscript for explanations).

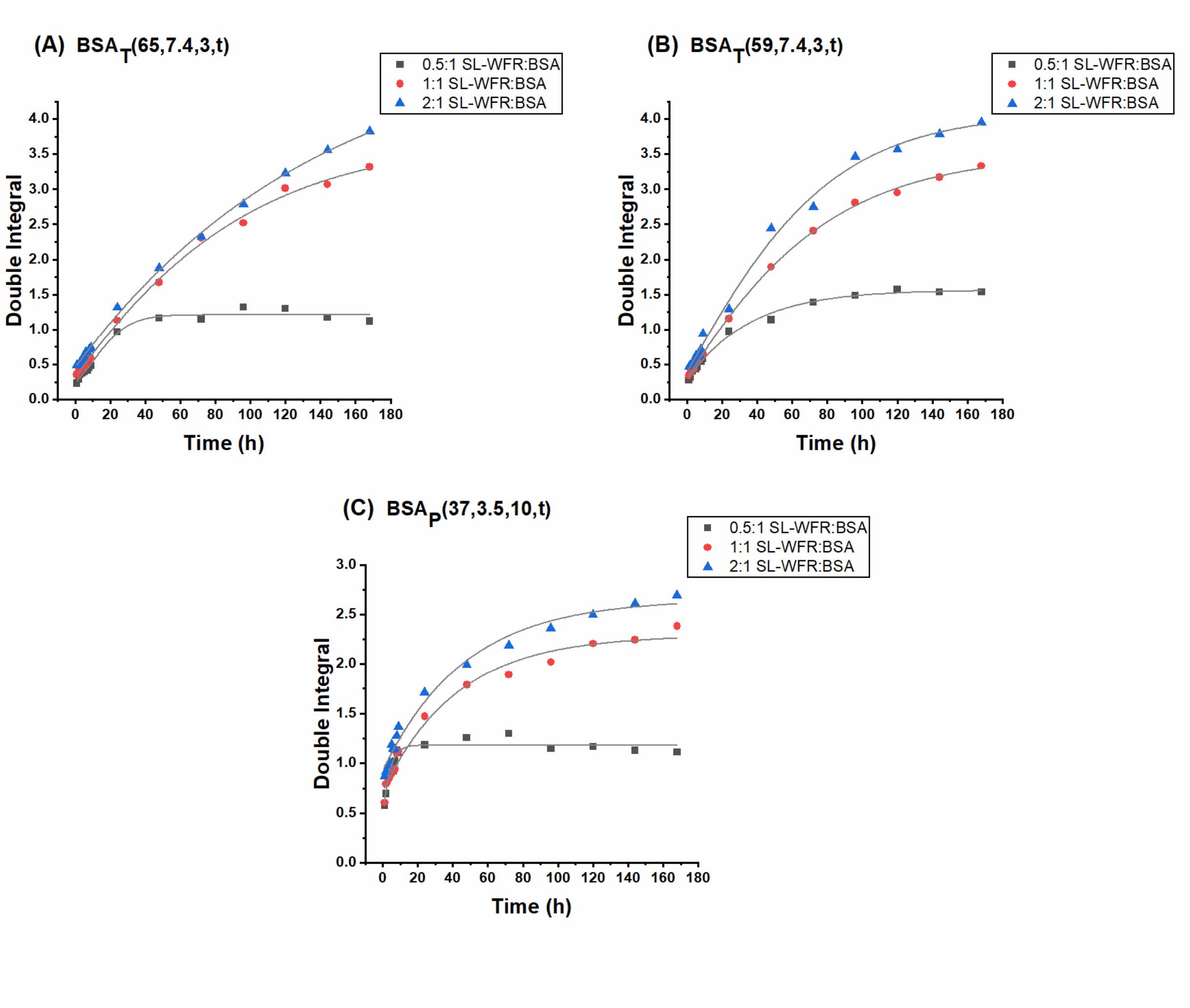

Figure S6. Release profiles of SL-WFR loaded BSA hydrogels with 0.5:1, 1:1 and 2:1 SL-WFR: BSA molar ratios (**A**) BSA_T(65, 7.4, 3) hydrogel, (**B**) BSA_T(59, 7.4, 3) hydrogel and (**C**) BSA_P(37, 3.5, 10) hydrogel. The curves are fitted.

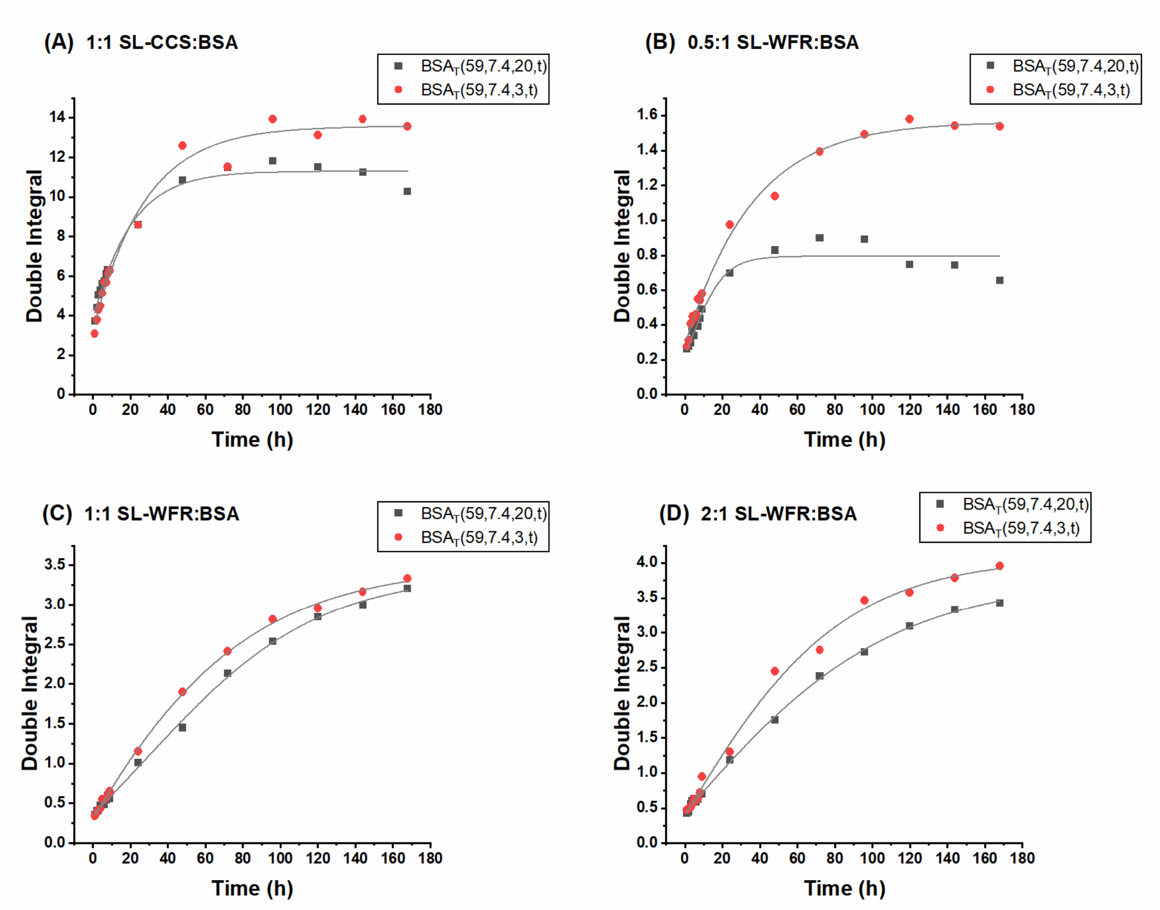

Figure S7. Release profiles of SL-CCS and SL-WFR loaded BSA hydrogels prepared with heat induced method at 59 °C with two different incubation times of 3 and 20 min at a (**A**) 0.5:1 SL-CCS: BSA molar ratio, (**B**) 0.5:1 SL-WFR: BSA molar ratio, (**C**) 1:1 SL-WFR: BSA molar ratio and (**D**) 2:1 SL-WFR: BSA molar ratio.

The direct effect of incubation time on release rate from hydrogels loaded with SL-CCS and SL-WFR is shown in Figure S7. We find that increasing the incubation time from 3 to 20 min slightly decrease the release rate. As it was explained in rheological characterization section, increasing the incubation time can result in more mechanically robust hydrogels, which have lower release rate.

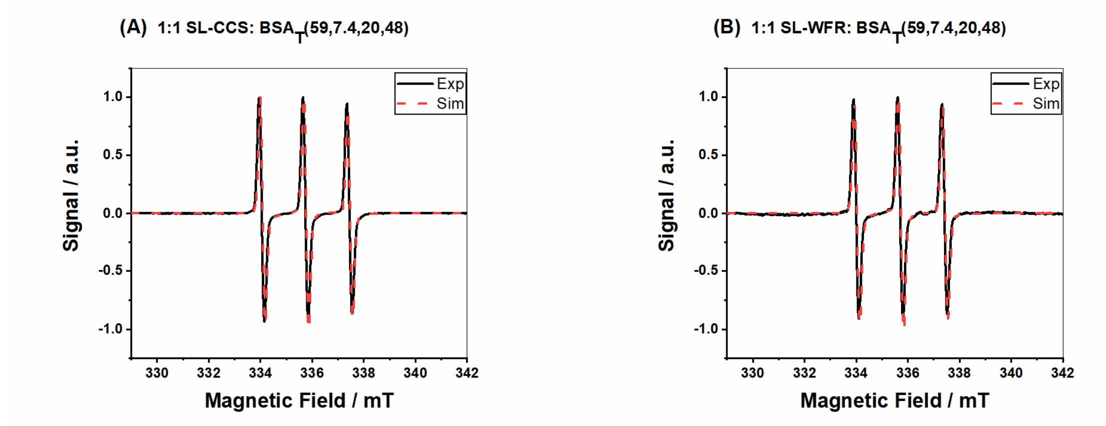

Figure S8. EPR spectra of release test after 48 h for SL-drugs loaded BSA hydrogels at a 1:1 molar ratio (**A**) SL-CCS: BSA_T(59, 7.4, 20, 48) and (**B**) SL-WFR: BSA_T(59, 7.4, 20, 48).

Table S3. Percentage of released components in different states gained from spectral simulation of Figure S8.

Figure	Percentage (bound, intermediate and free)
	**0**
A	**20**
	**80**
	**0**
B	**26**
	**74**

Figure S8 displays EPR spectra of release test after 48 h from hydrogels loaded with SL-CCS and SL-WFR prepared by heat induced method at 59 °C with 20 minutes of incubation time. As can be seen in Table S3 which shows the results obtained from simulation of EPR spectra in Figure S8, the highest fraction of released components in PBS is related to freely tumbling SL-CCS or SL-WFR, the small percentage shows intermediate rotational motion and there is no sign of strongly immobilized SL-pharmaceuticals to BSA in release medium.

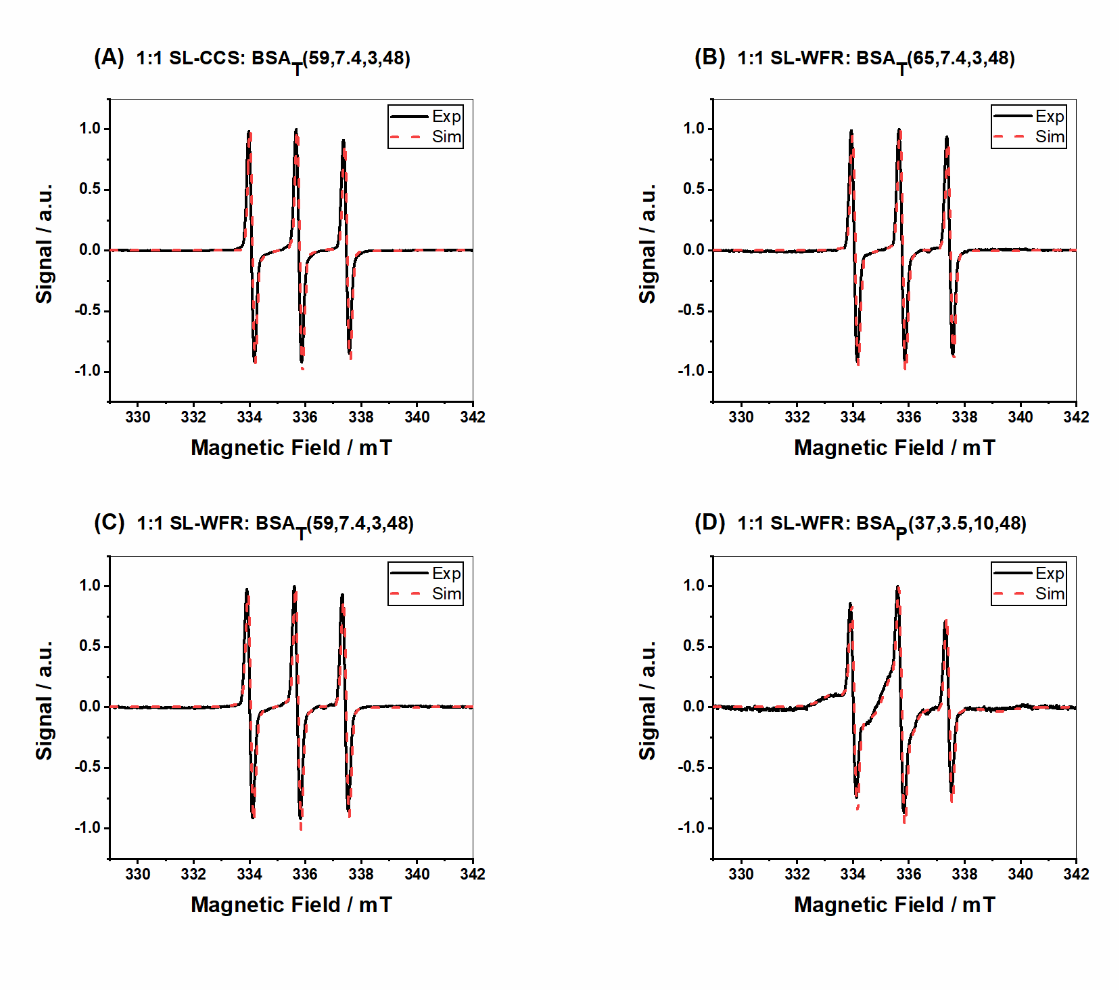

Figure S9. EPR spectra of release test after 48 hours for SL-drugs loaded BSA hydrogels at a 1:1 molar ratio (**A**) SL-CCS: BSA_T(59, 7.4, 3, 48), (**B**) SL-WFR: BSA_T(65, 7.4, 3, 48), (**C**) SL-WFR: BSA_T(59, 7.4, 3, 48) and (**D**) SL-WFR: BSA_T(37, 3.5, 10, 48).

Table S4. Percentage of released components in different states gained from spectral simulation of Figure S9.

Figure	Percentage (bound, intermediate and free)
**A**	0<br>25%<br>75%
**B**	0<br>68%<br>32%
**C**	0<br>64%<br>36%
**D**	57%<br>26%<br>16%

Figure S9 displays EPR spectra of release test after 48 h from hydrogels loaded with SL-CCS and SL-WFR prepared by heat and pH induced method with lower incubation times (3 min for thermally induced hydrogels and 10 min for electrostatically triggered gels). By comparing the results from Table S4 with Tables S2 and 2 (in the main manuscript), we can see the effect of incubation time on the released components. It is found that lowering the incubation time leads to the release of more intermediately bound SL-drug to BSA in case of heat induced gels and intermediately and strongly bound SL-pharmaceuticals to BSA for pH induced hydrogels.

Dynamic light scattering (DLS) measurements

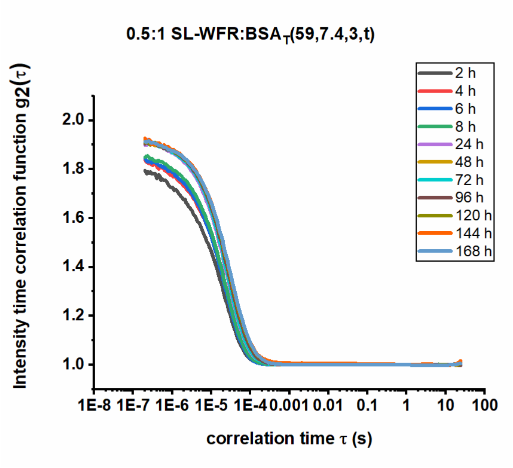

Figure S10. Intensity time correlation functions for 0.5:1 SL-WFR:BSA$_T$(59, 7.4, 3) molar ratio during different release times.

Figure S10 shows intensity time correlation function during release time from BSA hydrogel with SL-WFR at a 0.5:1 molar ratio prepared by heat induced method at 59 °C. It seems that y-intercept value of the autocorrelation function is slightly lower during the first 8 hours which shows a decrease in the scattering intensity. Probably during the first hours the release rate is slow while at later release times the scattering intensity increases. However, all of the curves decay almost at the same time indicating the release of at least one component with the same size over release periods.

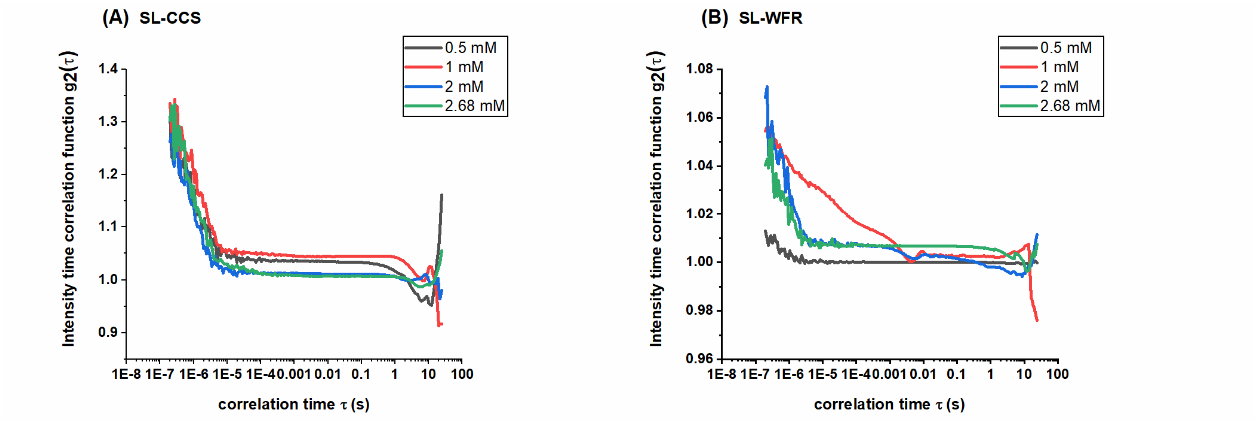

Figure S11. Intensity time correlation functions for different concentrations of (**A**) SL-CCS diluted with 10× PBS and (**B**) SL-WFR diluted with 10× PBS.

Figure S11 shows that there is no pronounced autocorrelation function for SL-drugs diluted with PBS which indicates the absence of well-defined structures.

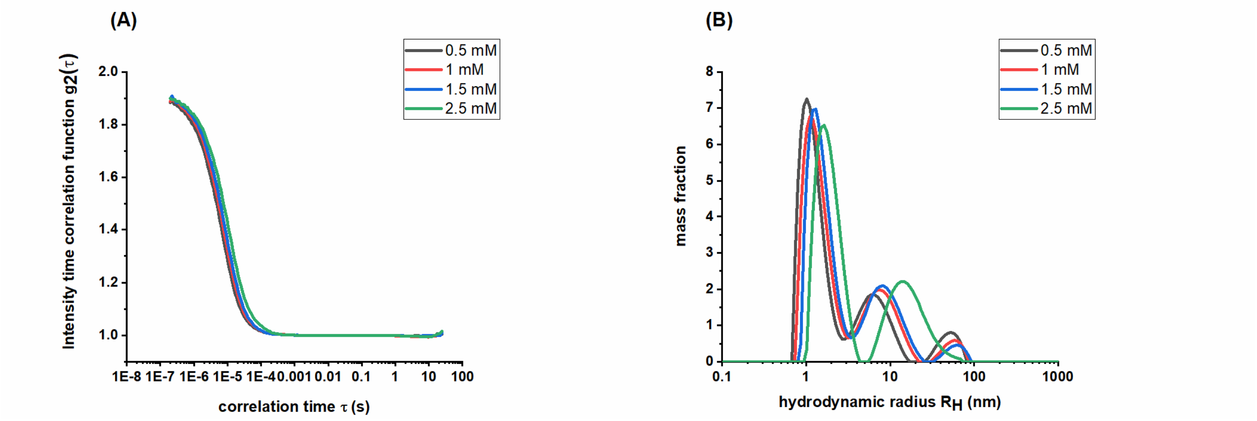

Figure S12. (**A**) Intensity time correlation functions for different concentrations of BSA solution diluted by 10× PBS and (**B**) hydrodynamic radius of different concentrations of BSA solution diluted by 10× PBS.

Figure S12A illustrates pronounced autocorrelation functions and high scattering intensities, indicating the presence of highly defined particles in solution. Figure S12B shows that there are dimers and oligomers of BSA which are below 100 nm in solution.

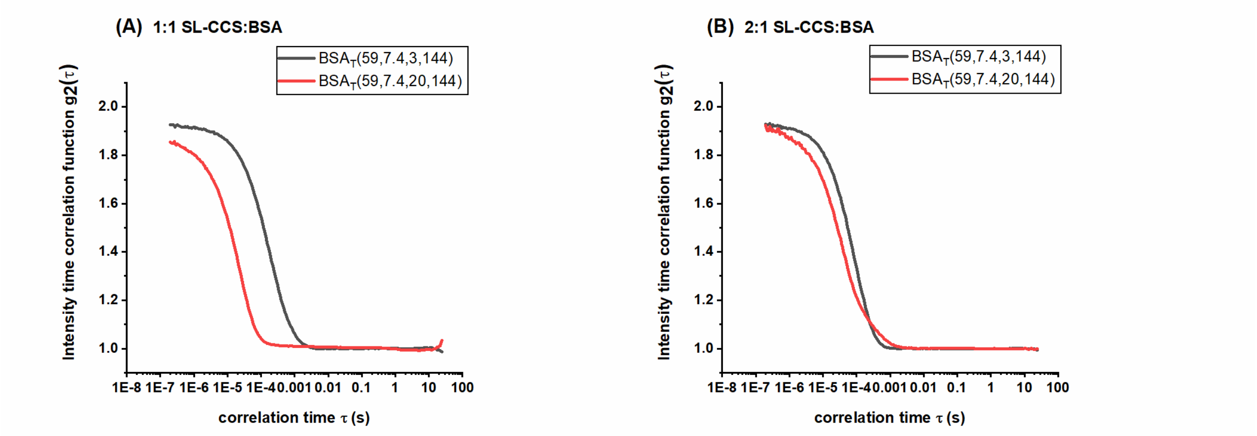

Figure S13. Intensity time correlation functions of (**A**) SL-CCS: BSA$_T$(59, 7.4, 3, 144) and SL-CCS: BSA$_T$(59, 7.4, 20, 144) at a 1:1 molar ratio and (**B**) SL-CCS: BSA$_T$(59, 7.4, 3, 144) and SL-CCS: BSA$_T$(59, 7.4, 20, 144) at a 2:1 molar ratio.

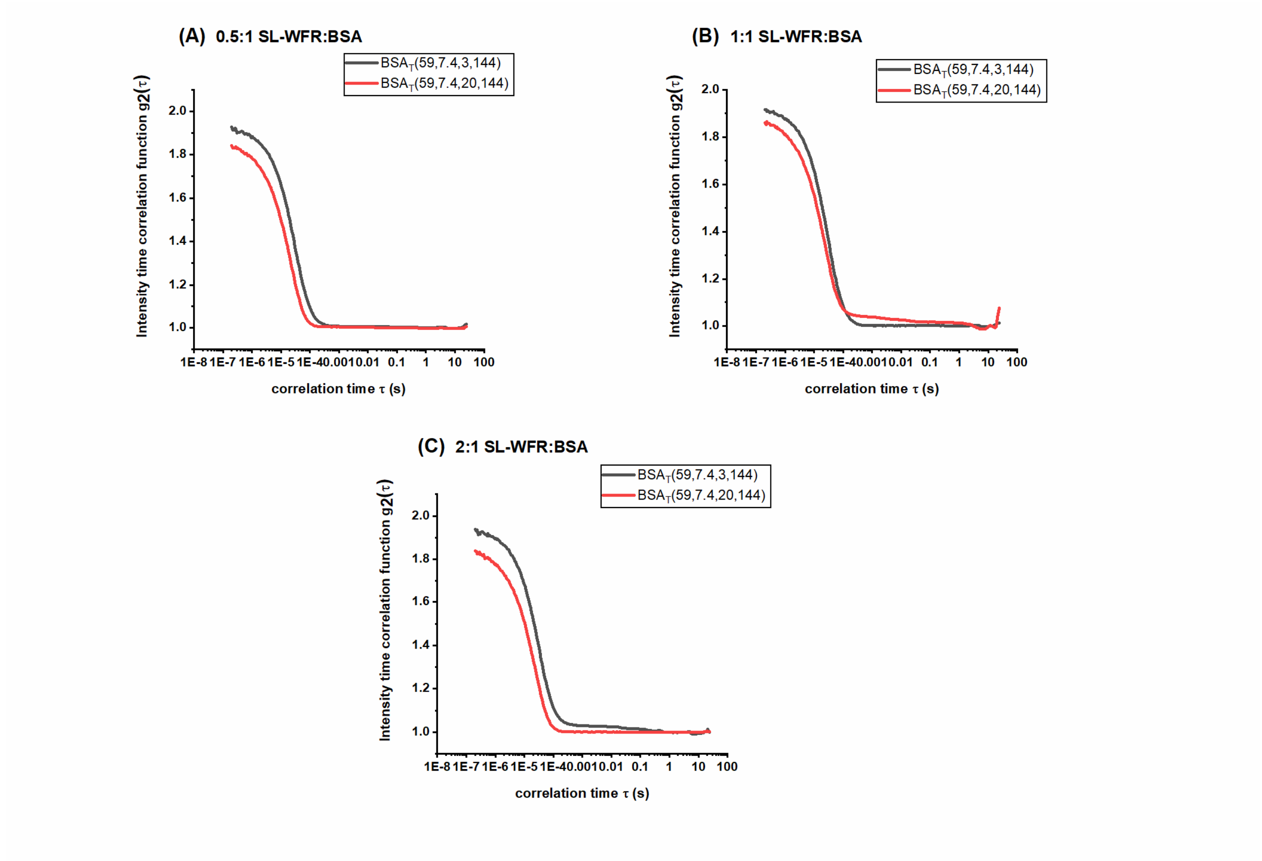

Figure S14. Intensity time correlation functions of (**A**) SL-WFR: BSA$_T$(59, 7.4, 3, 144) and SL-WFR: BSA$_T$(59, 7.4, 20, 144) at a 0.5:1 molar ratio, (**B**) SL-WFR: BSA$_T$(59, 7.4, 3, 144) and SL-WFR: BSA$_T$(59, 7.4, 20, 144) at a 1:1 molar ratio and (**C**) SL-WFR: BSA$_T$(59, 7.4, 3, 144) and SL-WFR: BSA$_T$(59, 7.4, 20, 144) at a 2:1 molar ratio.

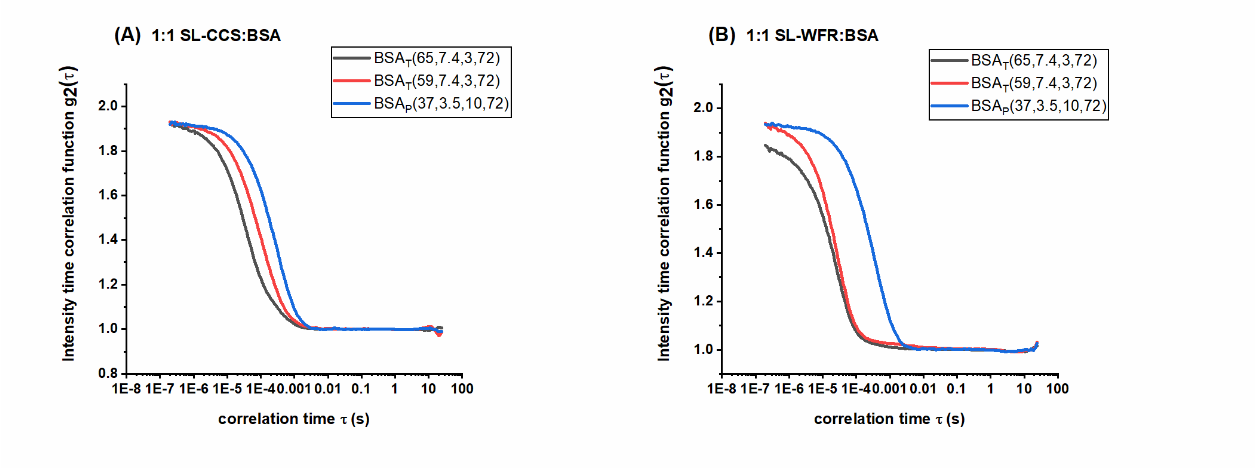

Figure S15. Intensity time correlation functions for (**A**) SL-CCS: BSA$_T$(65, 7.4, 3, 72), SL-CCS: BSA$_T$(59, 7.4, 3, 72) and SL-CCS: BSA$_P$(37, 3.5, 10, 72) at a 1:1 molar ratio and (**B**) SL-WFR: BSA$_T$(65, 7.4, 3, 72), SL-WFR: BSA$_T$(59, 7.4, 3, 72) and SL-WFR: BSA$_P$(37, 3.5, 10, 72) at a 1:1 molar ratio.
